# Delineating Astrocytic Cytokine Responses in a Human Stem Cell Model of Neural Trauma

**DOI:** 10.1089/neu.2019.6480

**Published:** 2019-12-11

**Authors:** Eric Peter Thelin, Claire E. Hall, Giulia E. Tyzack, Arvid Frostell, Susan Giorgi-Coll, Aftab Alam, Keri L.H. Carpenter, Jamie Mitchell, Tamara Tajsic, Peter J. Hutchinson, Rickie Patani, Adel Helmy

**Affiliations:** ^1^ Division of Neurosurgery, University of Cambridge, Cambridge, United Kingdom.; ^2^ Department of Clinical Neuroscience, Karolinska Institutet, Stockholm, Sweden.; ^3^ Department of Molecular Neuroscience, Institute of Neurology, University College London, London, United Kingdom.; ^4^ Wolfson Brain Imaging Centre, Department of Clinical Neurosciences, University of Cambridge, Cambridge, United Kingdom.; ^5^ The Francis Crick Institute, London, United Kingdom.

**Keywords:** cytokine exposure, human iPSC-derived astrocytes, IL-1β, IL-4, IL-6, IL-10, *in vitro*, TBI, TNF

## Abstract

Neuroinflammation has been shown to mediate the pathophysiological response following traumatic brain injury (TBI). Accumulating evidence implicates astrocytes as key immune cells within the central nervous system (CNS), displaying both pro- and anti-inflammatory properties. The aim of this study was to investigate how *in vitro* human astrocyte cultures respond to cytokines across a concentration range that approximates the aftermath of human TBI. To this end, enriched cultures of human induced pluripotent stem cell (iPSC)-derived astrocytes were exposed to interleukin-1β (IL-1β) (1–10,000 pg/mL), IL-4 (1–10,000 pg/mL), IL-6 (100–1,000,000 pg/mL), IL-10 (1–10,000 pg/mL) and tumor necrosis factor (TNF)-α (1–10,000 pg/mL). After 1, 24, 48 and 72 h, cultures were fixed and immunolabeled, and the secretome/supernatant was analyzed at 24, 48, and 72 h using a human cytokine/chemokine 39-plex Luminex assay. Data were compared to previous *in vitro* studies of neuronal cultures and clinical TBI studies. The secretome revealed concentration-, time- and/or both concentration- and time-dependent production of downstream cytokines (29, 21, and 17 cytokines, respectively, *p*<0.05). IL-1β exposure generated the most profound downstream response (27 cytokines), IL-6 and TNF had intermediate responses (13 and 11 cytokines, respectively), whereas IL-4 and IL-10 only led to weak responses over time or in escalating concentration (8 and 8 cytokines, respectively). Notably, expression of IL-1β, IL-6, and TNF cytokine receptor mRNA was higher in astrocyte cultures than in neuronal cultures. Several secreted cytokines had temporal trajectories, which corresponded to those seen in the aftermath of human TBI. In summary, iPSC-derived astrocyte cultures exposed to cytokine concentrations reflecting those in TBI generated an increased downstream cytokine production, particularly IL-1β. Although more work is needed to better understand how different cells in the CNS respond to the neuroinflammatory milieu after TBI, our data shows that iPSC-derived astrocytes represent a tractable model to study cytokine stimulation in a cell type-specific manner.

## Introduction

Globally, traumatic brain injury (TBI) is one of the most common causes of acquired disability and is associated with an extensive morbidity and mortality, with immeasurable subsequent costs for the patient, carers, and society.^1,2^ Although the initial impact is often detrimental, it also initiates different injury cascades because of impaired cerebral oxygen supply, perfusion mismatch, and metabolic dysfunction, causing a cytotoxic milieu for surviving cells in the central nervous system (CNS), which may lead to additional injuries.^3^ Strong evidence suggests that these processes are driven in part by neuroinflammatory reactions, primarily by the innate immune system.^4–6^ These inflammatory pathways are mediated predominantly through cytokine and chemokine interactions, which, after TBI, seem to act in a temporal and dose-dependent manner.^7,8^ Although the initial, primarily pro-inflammatory response has been suggested to have detrimental properties, it is now clear that the inflammatory reactions following TBI present both potentially harmful and beneficial effects in the injured brain.^9^ There is evidence that this inflammatory response is produced locally within the CNS following TBI,^7,10^ but that systemically recruited inflammatory cells may play a role as well.^11^ Although microglia have been described as the primary mediator of inflammation in the CNS,^12^ there is a growing interest in the immunocompetence of other cells in the CNS.^13^ Astrocytes, which are supportive glial cells, are increasingly recognized to possess properties similar to those of microglia/CNS macrophages. Astrocytes have been shown to exert both pro- and anti-inflammatory properties (termed A1/A2, respectively, following the taxonomy of microglial counterparts).^14–16^ The A1-phenotype, generated by exposure to (microglia-derived) tumor necrosis factor (TNF)-α, interleukin (IL)-1α, and complement proteins, lose several crucial homeostatic functions seen in normal astrocytes (including phagocytosis and synaptogenesis), becoming extensively neurotoxic to surrounding cells.^16^ Although A1 has been suggested to play a detrimental role in auto-immune diseases such as multiple sclerosis (MS),^16^ there is little knowledge about phenotypic polarization across this aforementioned A1/A2 spectrum in TBI pathophysiology. Moreover, the cytokine concentrations used to induce the astrocytic response in previous studies were 30 and 30,000 times higher for IL-1α and TNF, respectively, than levels seen in human brain extracellular fluid (ECF) following TBI.^7,16^

We have previously shown that enriched neuronal cultures exposed to pro-inflammatory cytokines in concentrations seen after TBI, especially IL-6 and TNF, produce a downstream cytokine response.^17^ Choi and coworkers have analyzed the cytokine secretome profile of human embryonal cultured astrocytes following relatively high levels of IL-1β and TNF in combination, and noticed a change in cytokine patterns after 24 h.^18^ Although this approach is noteworthy, we believe it is also important to study these responses in a time-resolved manner, with cytokine concentrations empirically measured in the injured brain. Further, investigating cytokine responses following stimulation with putative anti-inflammatory cytokines is also of great relevance in this context. Against this background, we aimed to elucidate cell-autonomous astrocyte responses in an enriched culture that had no prior exposure to other cells (e.g., microglia) that may “prime” the astrocytes.^16^ In order to achieve this, we reasoned that human- induced pluripotent stem cell (iPSC)-derived astrocytes would be the optimal approach.

Therefore, the aim of the current study was to investigate how enriched astrocyte cultures respond to pro- (IL-1 β, IL-6, and TNF) and anti-inflammatory (IL-4 and IL-10) cytokines at concentrations found in the aftermath of human TBI, by analyzing downstream cytokine generation. We also wanted to study viability of the cell cultures, compare the astrocyte secretome with our previous findings in enriched neuronal cultures, and determine how these *in vitro*-generated cytokine responses compared in temporal profile and concentrations with what is seen in human TBI *in vivo*.

## Methods

### Human iPSC-derived astrocytes

Astrocyte differentiation was conducted using our previously published protocol.^19^ Briefly, iPSCs were first differentiated from neuroepithelium by plating to 100% confluency in chemically defined medium consisting of DMEM/F12 Glutamax, Neurobasal, L- glutamine, N2 supplement, non-essential amino acids, B27 supplement, β-mercaptoethanol (all from Life Technologies) and insulin (Sigma). Treatment with small molecules from day 0 to day 7 was as follows: 1 μM dorsomorphin (Millipore), 2 μM SB431542 (Tocris Bioscience), and 3 μM CHIR99021 (Miltenyi Biotec). On day 8, the neuroepithelial layer was enzymatically dissociated using dispase (GIBCO, 1 mg/mL), plated onto laminin-coated plates and next patterned for 7 days with 0.5 μM retinoic acid and 1μM Purmorphamine, followed by further 4 days with 0.1 μM Purmorphamine. To promote the gliogenic switch, neural precursors were then subjected to a propagation phase (> 60 days) with 10 ng/mL FGF-2 (Peprotech) before terminal differentiation for a minimum of 2 weeks in 10 ng/mL bone morphogenetic protein (BMP)4 and 10 ng/mL leukemia inhibitory factor (LIF), allowing the generation of high-purity astrocytic cultures (> 95% astrocytes) as previously described.^20^ Using this protocol, we have in previous articles shown that these astrocytes are similar to those of human origin using RNAseq and immunocytochemistry (showing that they express >90% of the astrocytic markers glutamate aspartate transformer [GLAST], glutamate transporter [GLT]-1, glial fibrillary acidic protein [GFAP], and aldehyde dehydrogenase 1 family member L1 [ALDH1L1]) as well as functionally characterizing these astrocytes by analyzing responses to physiological calcium stimuli (adenosine triphosphate [ATP]) and cytokine stimulation assays.^19–21^

This study utilized two control lines in total; one commercially available, named CTRL 2 from Coriell Institute for Medical Research, Camden, NJ (cat. number ND41866*C) and the other (CTRL 1) reprogrammed in-house from donor fibroblasts.^22^ This line was obtained from the laboratory of Dr. Selina Wray as a gift.

### Enriched motor neuron (MN) culture preparation

iPSCs were maintained on Geltrex (Life Technologies) with Essential 8 Medium media (Life Technologies), and passaged using EDTA (Life Technologies, 0.5mM). All cell cultures were maintained at 37°C and 5% carbon dioxide. MN differentiation was performed using an adapted version of a previously published protocol (Hall et al., 2017). Briefly, iPSCs were first differentiated from neuroepithelium by plating to 100% confluency in chemically defined medium consisting of DMEM/F12 Glutamax, Neurobasal, L- Glutamine, N2 supplement, non-essential amino acids, B27 supplement, β-mercaptoethanol (all from Life Technologies) and insulin (Sigma). Treatment with small molecules from day 0 to day 7 was as follows: 1 μM Dorsomorphin (Millipore), 2 μM SB431542 (Tocris Bioscience), and 3 μM CHIR99021 (Miltenyi Biotec). On day 8, the neuroepithelial layer was enzymatically dissociated using dispase (GIBCO, 1 mg/mL), plated onto laminin coated plates and next patterned for 7 days with 0.5 μM retinoic acid and 1μM Purmorphamine. At day 14, MN precursors were treated with 0.1μM Purmorphamine for a further 4 days before being terminally differentiated in 0.1 μM Compound E (Enzo Life Sciences) to promote cell cycle exit.

### Cytokine induction

Each of the two experimental blocks was technically duplicated (i.e., two culture wells). Each culture well was further technically duplicated (i.e., two supernatants isolated per time point) as described. This resulted in 18 experimental samples (2 technical repeats for each of 3 different inducing cytokine concentrations at 3 different time points) and three unstimulated control samples (technical repeats).

Human recombinant IL-1β (#11457756001), IL-4 (#I4269), IL-6 (#I1395), IL-10 (#IL010), and TNF (#T6674) (all from Merck, Kenilworth, NJ) were each sourced as lyophilized powder, which was reconstituted according to the manufacturer's instructions. Serial dilution of a stock solution generated the final concentrations seen in Table 1. Each cytokine was used at three concentrations covering the range of concentrations seen in human microdialysis studies, adjusted for relative recovery determined *in vivo.*^7^ We also included somewhat higher doses to adjust for an early phase of injury before microdialysis monitoring was instituted (Table 1). The cytokine solution (10 μL) was added to 990 μL of culture within each well to make up the desired final concentration within 1 mL at the time of medium replacement. Cell cultures were then returned to an incubator.

**Table 1.** Cytokine Concentrations Used to Induce a Downstream Cytokine Response

Concentration	IL-1b	IL-6	TNF	IL-4	IL-10
Range seen physiologically	0.02–21	0.15–4990	0.05–23	0.1–37	0.8–173
Low	1 pg/mL	100 pg/mL	1 pg/mL	1 pg/mL	1 pg/mL
Medium	100 pg/mL	10,000 pg/mL	100 pg/mL	100 pg/mL	100 pg/mL
High	10,000 pg/mL	1,000,000 pg/mL	10,000 pg/mL	10,000 pg/mL	10,000 pg/mL

IL, interleukin; TNF, tumor necrosis factor.

Cytokine concentrations used to stimulate the enriched astrocyte cultures. The concentrations are within range to what is seen in the extracellular space as seen in the aftermath of TBI as measured by microdialysis.^1^

1. Helmy, A., Guilfoyle, M.R., Carpenter, K.L., Pickard, J.D., Menon, D.K. and Hutchinson, P.J. (2014). Recombinant human interleukin-1 receptor antagonist in severe traumatic brain injury: a phase II randomized control trial. J Cereb Blood Flow Metab 34, 845-851.

### Sample collection and storage

Supernatant (60 μL) was taken from each cell culture well at the given time points (24 h, 48 h, and 72 h), including control lines (*n* = 3 technical repeats). The cells were collected at 1 h, 24 h, 48 h, and 72 h following addition of the respective cytokine and from untreated cultures (*n* = 3 per time point). Samples were stored at -80°C until analysis.

### Cytokine analysis

Each experimental condition/technical repeat was analyzed in duplicate (i.e., two samples per experimental cell culture well). The supernatants were analysed using the Procartaplex™ 39-PLEX, Human Cytokine/Chemokine 39 (Thermofisher, Waltham, MA) using the manufacturer's instructions as previously described,^17^ with overnight incubation. As described, the time points 0 (for control wells), 24, 48, and 72 h were analyzed. The plates were analyzed on a Luminex 200 platform (Luminex Corporation, Austin, TX). As per manufacture's recommendation, cytokine concentrations were calculated by reference to an eight-point five-parameter logistical standard curve for each cytokine.

### Immunocytochemistry

For immunocytochemistry, astrocytes were plated onto clear bottom 96 well plates (Falcon). These were fixed with 4% fresh paraformaldehyde for 10 min at room temperature before being washed three times with phosphate-buffered saline (PBS). Samples were then blocked for 1 h at room temperature with 0.3% Triton/PBS/5% goat serum before being incubated overnight with primary antibody in 0.1% Triton/PBS/5% goat serum at 4°C. Primary antibodies used were mouse monoclonal anti-GFAP (Clone G-A-5, 1:500, Sigma-Aldrich) and rabbit anti-cleaved caspase-3 (1:300, Cell Signaling Technology #9661). After three washes in PBS, a secondary antibody (goat anti-mouse, Alexa Fluor 488 or 568, 1:1000) in 0.1% Triton/PBS/5% goat serum was next applied for 1 h at room temperature. Nuclei were counterstained with DAPI (100 ng/mL).

Images were acquired using the automated Opera Phenix High-Content Screening System (Perkin Elmer). For automated image analysis, the Columbus Image Analysis System (Perkin Elmer) was used. Five non-overlapping fields per well were acquired, and two wells per condition (total >5000 cells) were analyzed.

### RNA extraction, sequencing, and analysis

Single-stranded RNAseq data was generated from iPSC-derived astrocytes and was analyzed alongside previously published RNAseq data from iPSC-derived MN.^19^ Both MN and astrocyte cultures were derived from the same control iPSC lines using two biological repeats in technical duplicate and were processed using the same workflow described subsequently. In addition to using two lines (from two different people), we additionally used two independent neural inductions from each of these lines, as we reasoned that this would allow us to incorporate both inter- and intra-patient variability while increasing the power by conducting a second experimental block for each line. Each of the two experimental blocks was technically duplicated (i.e., two culture wells).

A Promega Maxwell RSC simplyRNA cells kit including DNase treatment, alongside a Maxwell RSC instrument, was used for RNA extractions. A NanoDrop (Thermo Scientific, Waltham, MA) was used to assess RNA concentration and the 260/280 ratio, and an Agilent Bioanalyser (Agilent Technologies, Santa Clara, CA) was used to assess quality. RNA integrity (RIN) scores were >8 for all samples used in this work. RNAseq libraries were prepared using a Truseq stranded mRNA kit (Illumina) with 1 μg input RNA. The products were then purified and enriched with polymerase chain reaction (PCR) amplification to create the final cDNA libraries. Libraries were sent for high throughput sequencing, run for 75 cycles on a rapid flow cell, at the Institute of Neurology's next generation sequencing (NGS) core facility using the Hiseq2500. RNA-seq data were generated from poly(A)+ RNA. After removing the adapter sequences and quality checking the data using FastQC, the reads were initially aligned to ribosomal RNA sequences to filter out reads that might have come from ribosomal RNA contamination using bowtie2 (-v 0). The remaining reads were then aligned to the human genome (h19) using the splice aware aligner TopHat2 with default parameters. The absolute quantification of the genes was performed using HTSeqcount. The raw read count files obtained were then processed for unsupervised clustering analysis. The EdgeR package in R was used to generate normalized reads per kilobase of transcript (RPKM) values. For corresponding cytokine receptors for the inducing cytokines, these values were mean centralized and plotted using R version 3.2.3 (Table S1). All libraries used had <1% ribosomal RNA (rRNA), <1% mitochondrial DNA (mtDNA), >90% strandedness, and >70% exonic reads (data not shown).

### Clinical cytokine data from TBI patients

We extracted data from two previously published studies from our group describing the temporal profiles and concentrations of cytokines from severe TBI patients.^7,23^ In a pilot trial of IL-1 receptor antagonist treatment for TBI, we used brain cytokine concentrations from the placebo group.^23^ Demographic data for all patients are available in Table S2, including age, gender, admission Glasgow Coma Scale (GCS), and head computerized tomography (CT) verifiable injury as defined by the Marshall CT classification (Grades II–IV = diffuse and Grade VI = focal).^24^ As described in detail in the previous studies,^7,23^ patients were monitored for a maximum of 5 consecutive days and cytokine/chemokine samples were pooled from 6 h periods. Although there was some disparity in commencement of monitoring following ictus, all data have been adjusted to the trauma time to enable comparison with the *in vitro* experiments.

### Statistical analysis

The statistical program R, with the interface R-studio, was used for the analyses.^25^ The effect of each of the added cytokines (IL-1β, IL-4, IL-6, IL-10, and TNF) on the measured cytokines (39 cytokine panel) was analyzed using a two way mixed analysis of variance (ANOVA) (to compare concentrations of each cytokine analyzed). The time at which the sample was taken (“time”; 24 h, 48 h, 72 h) was used as the repeated measure (within-subject) variable. The concentration of added cytokine (“concentration”; untreated, and three concentrations of added cytokine shown in Table 1) was used as the independent (between-subject) variable. A “time and concentration” relationship was also generated to ascertain whether time and concentration as an interactive model affected downstream cytokine generation.

For the immunocytochemistry analyses, data are presented as mean ± standard deviation (SD) from two technical replicates. Two way ANOVA with Dunnett's multiple comparison tests was performed.

Descriptively, we compared the temporal profiles of cytokine release as well as concentrations *in vivo*, including from enriched neuronal cultures *in vitro*,^17^ from previous studies and compared it with our results.^7,23^

## Results

### RNAseq data of iPSC-derived astrocyte and MN cultures

**FIG. 1.**
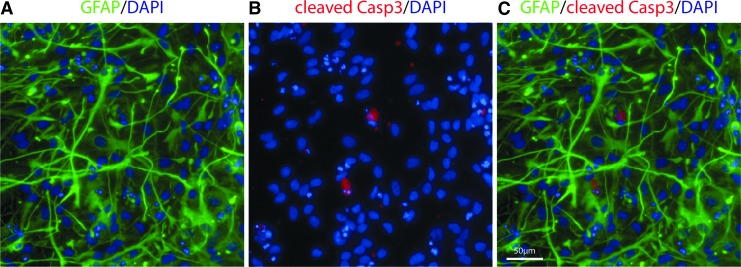
Immunocytochemistry illustrating the enriched astrocytic cell cultures. **(A)** A co-staining of DAPI and glial fibrillary acidic protein (GFAP) showing a representative image of the cultures. **(B, C)** Highlights the presence of cleaved Caspase 3 a sign of apoptosis. Scale bar 50 μm. Color image is available online.

**FIG. 2.**
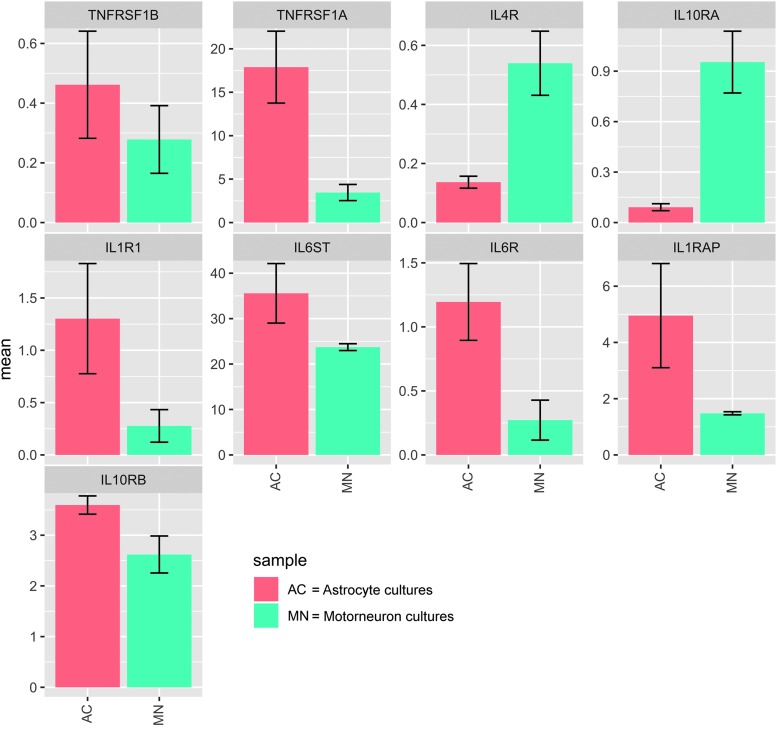
Bar plots illustrating a transcriptional analysis of cytokine receptor mRNA levels between unstimulated motor neuronal (MN) and astrocyte cultures (AC). Neurons were analyzed after 17 days of terminal differentiation, 3 technical repeats from each of 2 separate control lines. Astrocytes were analyzed 28 days after terminal differentiation, technical *n* = 6 (from two biological control lines, in triplicate). Y-axes are reads per kilobase of transcript (RPKM) per million mapped reads. Error bars represent mean and standard error of the mean (SEM). TNFRSF1B, tumor necrosis factor receptor superfamily 1B; TNFRSF1A, TNF receptor superfamily 1A; IL4R, interleukin-4 receptor; IL10RA, IL-10 receptor A; IL1R1, IL-1 receptor 1; IL6ST, IL-6 signal transducer; IL6R, IL-6 receptor; IL1RAP, IL-1 receptor accessory protein; IL10RB, IL-10 receptor B. Color image is available online.

We used our previously published extensively characterized method of astrogliogenesis,^19^ for which a representative image is provided in Figure 1A. Using RNAseq data we first analyzed the relative gene expression of selected cytokine receptors in both iPSC derived astrocytes and motor neurons (Table S1) (Fig. 2). These data show that astrocytes broadly exhibit increased expression of IL-1β, IL-6, and TNF, whereas the MN counterparts exhibit higher expression of IL-4 and IL-10 receptors, suggesting their competence to respond to specific cytokine stimulation.

### Temporal and concentration dependent effects of added cytokines to astrocyte cultures

We noticed downstream cytokine production using the predetermined, pathophysiologically relevant concentration ranges, with IL-1β (27 cytokines, *p* < 0.05) as the strongest inducer in a majority of cases (Table 2). IL-6 (13 cytokines) and TNF (11 cytokines) led to an intermediate level of downstream cytokine production, although fewer were detected if stimulated with IL-4 and IL-10 (8 cytokines each) (Tables 2 and 3). Figure S1 includes the ANOVA output for the experimental conditions showing a statistically significant response with increasing levels of cytokines as compared with unstimulated cultures.

**Table 2.** Downstream Cytokine Analysis of the Astrocyte Culture Secretome

	Time					Concentration					Time × concentration				
	IL-1b	IL-4	IL-6	IL-10	TNF	IL-1b	IL-4	IL-6	IL-10	TNF	IL-1b	IL-4	IL-6	IL-10	TNF
IFN-α	-	-	-	-	-	-	-	-	-	-	-	-	-	-	-
IL-23	-	-	-	-	-	-	-	-	-	-	-	-	-	-	-
MCP2	↑	-	-	-	-	↑	-	-	-	-	↑	-	-	-	-
MCP3	-	-	-	-	-	-	-	-	-	-	-	-	-	-	-
MIP3α	-	-	-	-	-	-	-	-	-	-	-	-	-	-	-
IL17A	-	-	-	-	-	-	-	-	-	-	-	-	-	-	-
IL-8	↑	↑	-	↑	↑	↑	-	-	↑	-	-	↑	↑	-	↑
MCP-1	-	-	-	-	-	-	-	-	-	-	-	-	-	-	-
MIP1α	↑	-	↑	-	-	↑	-	↑	-	-	-	-	↑	-	-
BAFF	↑	-	-	-	-	-	-	-	-	-	-	-	-	-	-
G-CSF	-	-	-	-	-	-	-	-	-	-	-	-	-	-	-
IFN-γ	-	-	-	-	-	-	-	-	-	-	-	-	-	-	-
IL-12p70	-	-	-	-	-	-	-	-	-	-	-	-	-	-	-
IL-1ra	-	-	-	-	-	↑	-	-	-	-	-	-	-	-	-
IL1α	↑	-	-	-	↑	↑	-	-	↑	-	↑	↑	↑	-	↑
MMP2	-	-	-	-	-	-	-	-	-	-	-	-	-	-	-
RANTES	-	-	-	-	-	↑	-	-	-	-	-	-	-	-	-
TIMP1	↑	-	↑	-	-	-	-	↑	-	-	-	-	-	-	-
BDNF	-	-	-	-	-	-	-	-	-	-	-	-	-	-	-
BLC/CXCL13	-	-	-	-	-	-	-	-	-	-	-	-	-	-	-
GRO-α	-	-	-	-	-	↑	-	-	-	-	↑	-	-	-	-
IP10	-	-	-	-	-	↑	-	-	-	-	-	-	-	-	-
MMP9	↑	↑	↑	↑	↑	-	-	-	-	-	-	-	-	-	-
TGF-α	-	-	-	-	-	-	-	-	-	-	-	-	-	-	-
TNF-RI	-	-	-	-	-	-	-	-	-	-	-	-	-	-	-
VEGF-A	↑	↑	↑	↑	↑	-	↑	↑	-	-	-	↑	-	↑	↑
Eotaxin	-	-	-	-	-	↑	-	-	-	-	-	-	-	-	-
Fractalkine	-	-	-	-	-	↑	-	-	-	-	↑	-	-	-	-
M-CSF	-	↑	↑	↑	↑	↑	-	-	-	-	-	-	↑	-	-
MDC	-	-	-	-	↑	↑	-	-	-	-	-	-	-	-	↑
MIP1β	↑	-	-	-	-	↑	-	-	-	-	-	-	-	-	↑
VEGFD	-	-	-	-	-	↑	-	-	-	-	-	-	-	-	-
sCDL40	-	-	-	-	-	-	-	↑	-	-	-	-	-	-	-
Sum	9	4	5	5	6	14	1	4	2	0	4	3	4	1	5

The results are divided by analysis of variance (ANOVA) models with a significant change over time, concentration and time × concentration for each inducing cytokine as compared with baseline. The “↑” indicates that the specific cytokine induction produced a significant increased result for a specific cytokine in that model, whereas “-” indicates no significant increase. The sum of all significant increases is presented at the bottom of each column. A more detailed description of the data is available in Figure S1.

IFN, interferon; IL, interleukin; MCP, monocyte chemoattractant protein; MIP, macrophage inflammatory protein; BAFF, B cell activating factor; G-CSF, granulocyte colony-stimulating factor; MMP, matrix metallopeptidase; RANTES, regulated upon activation, normal T cell expressed, and secreted; TIMP, tissue inhibitor matrix metalloproteinase; BDNF, brain-derived neurotrophic factor; BLC, B lymphocyte chemoattractant; CXCL, C-X-C motif chemokine; GRO, growth-regulated oncogene; IP, interferon gamma-induced protein; TGF, transforming growth factor; TNF-RI, tumor necrosis factor receptor 1; VEGF, vascular endothelial growth factor; M-CSF, macrophage colony-stimulating factor; MDC, ; sCDL40, soluble CD40 ligand.

**Table 3.** Summary of Cytokine Induction

Cytokine	Cytokine induced		
added	Time	Concentration	Time × concentration (or time and concentration)
IL-1b	BAFF, TIMP1, MMP9, VEGF-A	IL-1ra, RANTES, IP10, Eotaxin, M-CSF, MDC, VEGF-D	MCP-2, IL-8, MIP1a, IL-1a, GROα, Fractalkine, MIP1b
IL-4	MMP9, M-CSF		IL-8, IL-1a, VEGF-A,
IL-6	MMP9	sCD40L	IL-8, MIP1a, IL-1a, TIMP1, VEGF-A, M-CSF
IL-10	MMP9, M-CSF	IL-1α	IL-8, VEGF-A
TNF	MMP9, M-CSF		IL-8, IL-1a, VEGF-A, MDC, MIP1b

Table of the cytokines induced by time, concentration, and time × concentration by stimulation of different cytokines.

IL, interleukin; TNF, tumor necrosis factor; BAFF, B cell activating factor; TIMP, tissue inhibitor matrix metalloproteinase; MMP, matrix metallopeptidase; VEGF, vascular endothelial growth factor; M-CSF, macrophage colony-stimulating factor; RANTES, regulated upon activation, normal T cell expressed, and secreted; IP, interferon gamma-induced protein; MDC, ; sCD40L, soluble CD40 ligand; MCP, monocyte chemoattractant protein; MIP, macrophage inflammatory protein; GRO, growth-regulated oncogene; TIMP, tissue inhibitor matrix metalloproteinase.

**FIG. 3.**
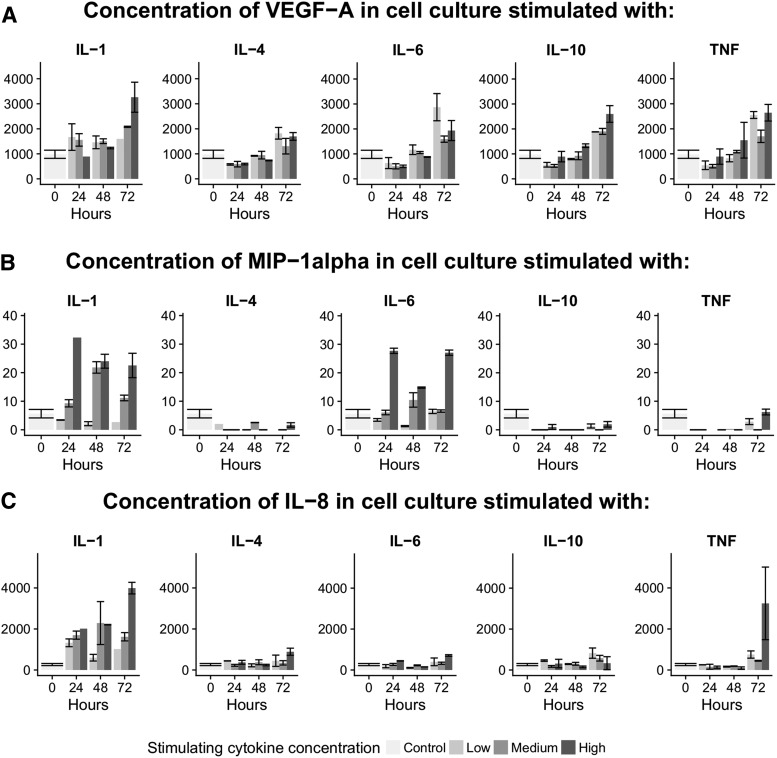
Examples of cytokines being significantly affected by **(A)** time (vascular endothelial growth factor [VEGF]-A), **(B)** concentration plotted against time (macrophage inflammatory protein [MIP]-1α), and (C) time × concentration (interleukin [IL]-8). Color image is available online.

We next considered the underlying principles of secondary cytokine release. These are exemplified by Figure 3 and summarized in Tables 2 and 3. Three patterns were evident. (1) A subset of cytokines was generated in a time-dependent fashion, without significance to escalating concentrations (29 cytokines, Fig. 3A) (Tables 2 and 3), including matrix metallopeptidase (MMP)-9 and macrophage colony-stimulating factor (M-CSF). (2) Another subset of cytokines was produced at a given concentration, but with no variation over time (21 cytokines, Fig. 3B) (Tables 2 and 3), including regulated upon activation, normal T cell expressed, and secreted (RANTES) and serum soluble CD40 ligand (sCD40L). (3) A third cytokine subset was observed that showed both a time and a concentration dependency (17 cytokines, Fig. 3C) (Tables 2 and 3), such as IL-8, vascular endothelial growth factor (VEGF)-A and IL-1α.

**FIG. 4.**
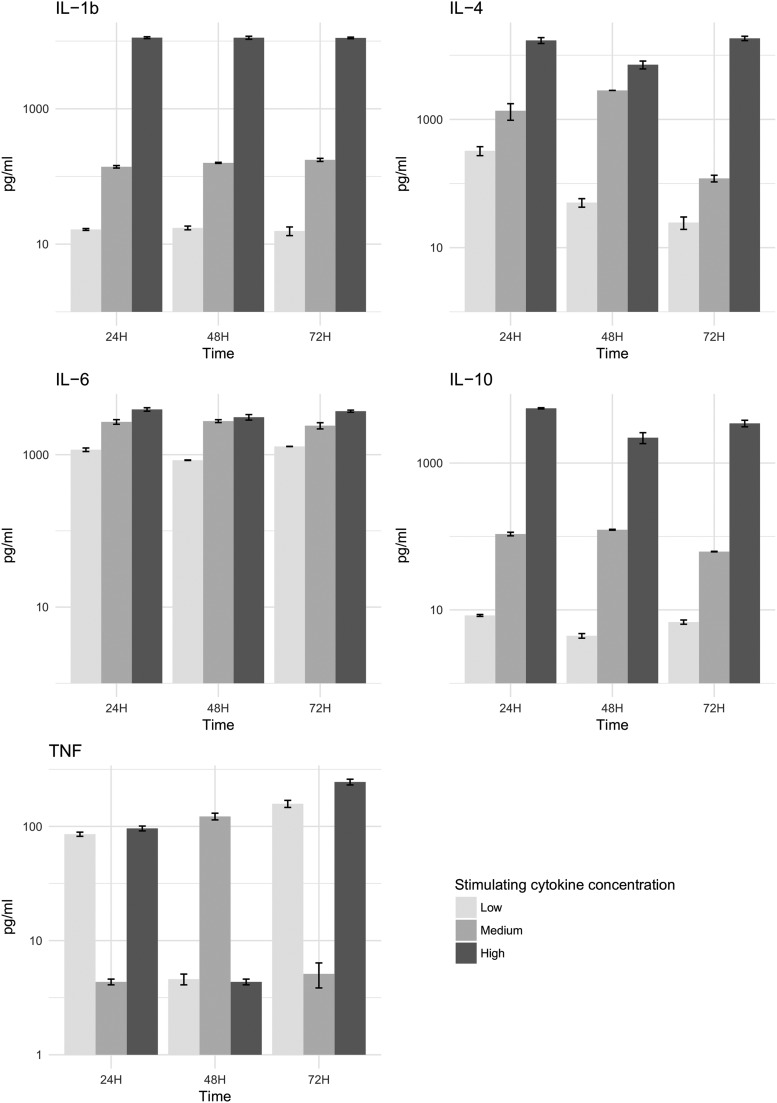
Validation of control cytokine concentrations from the Luminex assay. Error bars represent mean and standard error of the mean (SEM). Color image is available online.

The levels of exogenously added cytokines were maintained throughout all time points during the experiment (Fig. 4).

### Effect of secondary cytokine induction on astrocyte viability

#### Caspase-3 activation

We assessed the effects of cytokine exposure on astrocyte viability. Some cytokines induced a trend toward higher levels of caspase activity based primarily on either concentration (IL-4, IL-6, and IL-10) or time (TNF, IL-4, and IL-6) (Fig. S2). Indeed, there was a trend toward more caspase-3-activated cells (5–20%) across different pro-inflammatory cytokines, time points, and concentrations, whereas IL-4- and IL-10-exposed cultures had lower levels (5–10%) (Fig. S2). A representative figure of caspase activity can be seen in Figure 1B and C.

#### Pyknotic cell count

Looking at pyknotic cell count in the astrocytic cultures, there were no clear differences among stimulating cytokines, concentrations, or with cultures sampled at different time points, with an average of ∼30% pyknotic cells throughout (Fig. S3).

#### GFAP intensity

In comparison with the other cytokines, exposure to high concentrations of IL-1β decreased GFAP fluorescence intensity down to a maximum at 1500 arbitrary units per cell (Fig. S4). The other concentrations all had levels at ∼3000–4000 arbitrary units per cell. Interestingly, IL-4 had a distinct increase in GFAP intensity over time for all the concentrations tested.

### Cytokine temporal patterns *in vivo* versus astrocytes and neurons *in vitro*

In previous studies, we have characterized a time-resolved cytokine response following TBI *in vivo* as well as enriched neuronal cultures *in vitro* exposed to IL-1β, IL6, and TNF (Table 4).^7,17^ When comparing the secretome response with that of neuronal cultures, TNF was produced at higher concentrations in response to stimulation of the astrocytes (primarily IL-1β as can be seen in Table 2). Looking at *in vivo* human samples, there were several cytokines with similar temporal trends, albeit at much lower levels, which is probably because of the minimalistic approach, with only single cytokines added in the *in vitro* reductionist experiments.^7,17^ Notably, there were higher levels of IL-8 and G-CSF in the astrocyte cultures as compared with neuronal cultures. Furthermore, TNF production was relatively increased compared with neuronal cultures, being even higher than in *in vivo* concentrations.^7,17^

**Table 4.** Comparisons of Cytokine Levels *in Vivo* and Different *in Vitro* Models of Neuroinflammation

Cytokine	Time to peak* in vivo *following human TBI	Time to peak in* in vitro *astrocyte cultures	Mean (SEM) concentration* in vivo *(ECF) (pg/mL)	Mean (SEM) concentration neuronal* in vitro *(supernatant) (pg/mL)	Mean (SEM) concentration astrocyte* in vitro *(supernatant) (pg/mL)
TNF	<24h	up to 72h	3.0 (0.5)	0.6 (0.3)	84 (14)
IL8	up to 24h	up to 72h	1,816 (686)	87 (36)	387 (54)
MIP1α	up to 36h	up to 24h	84.8 (9.6)	19.5 (1.2)	8.9 (1.4)
sCD40L	up to 48h	48h	27.6 (3.0)	1.2 (0.4)	2.4 (0.8)
GRO	up to 48h	up to 72h	250 (42)	47 (7.0)	88 (16)
IL1β	up to 48h	up to 72h	3.9 (0.7)G	1.0 (0.1)	0.4 (0.05)
MIP1β	up to 60h	up to 72h	119 (16)	16.2 (2.1)	12.9 (4.2)
RANTES	up to 60h	up to 72h	79 (18)	21.3 (8.7)	14.8 (4.4)
IL1ra	24-72h	up to 72h	31 (7)	6.7 (0.6)	74 (16)
IL6	24-72h	up to 72h	622 (89)	195 (84)	84 (22)
G-CSF	24-72h	48h	380 (56)	1.8 (0.4)	104 (21)
IP10	24-72h	up to 72h	4,491 (618)	425 (247)	119 (29)
IL12p70	96-144h	48h	4.2 (0.4)	2.0 (0.2)	3.4 (0.2)
IL10	96-144h	up to 72h	18.9 (3.5)	5.6 (0.8)	3.1 (1.0)

Temporal release patterns and concentrations of some cytokines previously analyzed in the aftermath of human TBI (not adjusted for microdialysis relative recovery).^7,23^ Time to peak describes when the highest concentrations could be seen following trauma or cytokine induction. For comparison, both neuronal cultures from our previous experiment and the current one used the same concentrations of IL-1β, IL-6, and TNF stimulation from similar time points.^17^ For IL-1β, IL-6, and TNF concentrations, the added cytokine was removed, displaying only the downstream production generated by the other stimulating cytokines.

TBI, traumatic brain injury; SEM, standard error of the mean; ECF, extracellular fluid; TNF, tumor necrosis factor; IL, interleukin; MIP, macrophage inflammatory protein; sCD40L, soluble CD40 ligand; GRO, growth-regulated oncogene; RANTES, regulated upon activation, normal T cell expressed, and secreted; G-CSF, granulocyte colony-stimulating factor; IP, interferon gamma-induced protein,

## Discussion

We present the first study of human astrocyte cultures stimulated with five cytokines each employed separately, at a range of concentrations seen in the damaged parenchyma in the aftermath of TBI over time. By analyzing the secretome of human iPSC-derived astrocyte cultures, we noted increases of cytokines primarily driven by escalating levels of IL-1β, and, to a lesser extent, by TNF, IL-6, IL-4, and IL-10. Through RNAseq analysis of cytokine receptor expression, these findings are reinforced by specific receptor expression relative to enriched neuronal cultures. The escalating pathophysiological concentrations of cytokines to which the cultures were exposed had no significant detectable effect on cell viability, based on counts of nuclear pyknosis. However, the pro-inflammatory cytokines (IL-1β, IL-6, and TNF) resulted in higher degrees of caspase-3 activation than IL-4 and IL-10. In comparison with our previous studies in enriched neuronal cultures and human *in vivo* levels, the human enriched astrocyte cultures had a distinct secretome profile.

We stimulated human iPSC-derived astrocyte cultures using clinically relevant escalating concentrations of several cytokines, and studied the downstream cytokine production in the secretome over time. In a recent study, reactive and neurotoxic (A1) astrocytes were generated *in vitro* by culturing purified astrocytes for 6 days followed by 24 h of treatment with 3 ng/mL Il-1(α), 30 ng/mL TNF, and complement component 1q (C1q) 400 ng/mL.^16^ These A1 astrocytes lost many of the functions seen in normal astrocytes, and were shown to be highly neurotoxic if co-cultured with neurons.^16^ In comparison with our own experience of cytokine expression in the brain following TBI in humans,^7,23,26^ the levels of IL-1 and TNF are higher than those seen *in vivo*. Indeed, our highest level of TNF was only 33% (10 ng/mL) of that which was used previously,^16^ and although we did not test exactly the same cytokines, high concentrations and the potent stimulators used in this aforementioned article^16^ make direct comparisons with our findings challenging. It is possible that higher local concentrations of cytokines such as TNF could be present at a local cellular level, acting in a paracrine fashion, which cannot be accurately captured with techniques (such as cerebral microdialysis) available in human studies.^7,27^

Although it is common to only look at a few downstream cytokines using enzyme-linked immunosorbent assays (ELISAs), another study used Luminex in order to better characterize the inflammatory secretome of astrocytes. Choi and colleagues looked at the secretome from human enriched astrocyte cultures derived from dissociated fetal brain (15 weeks' gestation) exposed to a mixture of 10 ng/mL IL-1β and 10 ng/mL TNF (the same as our highest concentrations for both) after 24 h.^18^ Following stimulation, they noted upregulation of IL-1β, IL-1ra, TNF, interferon gamma-induced protein 10 (IP-10) (C-X-C motif chemokine 10 [CXCL10]), macrophage inflammatory protein 1-alpha (MIP-1α) (chemokine [C-C motif] ligand 3 [CCL3]), RANTES (CCL5), soluble intercellular adhesion molecule-1 (sICAM-1) and C5. Some of these cytokines were also seen on our study (almost exclusively in wells stimulated with IL-1β), but not nearly as many as in the study by Choi and colleagues. This is probably because they combined relatively high levels of inducing cytokines, which clearly seems to have a cumulative effect (distinct from the anti-inflammatory cytokines IL-4 and IL-10). Their conclusion, by looking at functional networks of downstream cytokines, was that the response by IL-1β and TNF seems to be driven primarily by nuclear factor (NF)-κB.^18^ Our choice of using iPSC-derived astrocytes also creates a more enriched and naïve culture without exposure to microglial – or indeed other heterotypic intercellular – stimulation, which could explain our different results.^28^ Additionally, iPSC-derived astrocytes are regionally defined. In primary cultures it is often not possible to retrieve enough astrocytes from a specific region, making pooling necessary. Further, these cells likely lose their regional identity *ex vivo,* unlike iPSC-astrocytes, which retain their regional identity even after long periods of culture.^28^

Consistent with our study, Santos and colleagues used stimulated iPSC astrocyte cultures (as well as astrocyte cultures from embryonal origin) with 10 ng/mL IL-1β and 50 ng/mL TNF for 5 h.^29^ They looked specifically at IL-6 and IL-8, and noted increased production of predominantly IL-8, and to a lesser extent IL-6, which is consistent with our data. Likewise, they did not report substantial differences in cell survival in cultures following stimulation, demonstrating that the iPSC astrocyte reductionist model can faithfully recapitulate key aspects of cytokine stimulation. They also studied the transcriptomic response to IL-1β stimulation, and found distinct upregulation of genes controlling for inflammatory response, immune response, chemokine activity, and cytokine activity.^29^ Moreover, similar to Liddelow and coworkers,^16^ they noted that these stimulated astrocytes affected neuronal morphology and viability.^29^ Recently, Perriot and coworkers stimulated human iPSC astrocyte cultures with IL-1β (10 ng/mL), IL-6 (100 ng/mL) and TNF (10 ng/mL), similar to our highest levels of stimulation.^30^ Consistent with our study, they noted the greatest increase of cytokines in the secretome after IL-1β stimulation, followed by TNF and IL-6. Because they did not perform a dose escalation study, and did not look at the secretome over time, comparisons are difficult, although many of the analyzed cytokines reached levels seen in our study.^30^

The response to TNF in our study was mixed, and the escalating concentration of TNF did not coherently overlap when the secretome was later analyzed (Fig. 3). Aloisi and coworkers performed human embryonic brain cultures (8–9 weeks of gestation) and induced them with IL-1β and TNF in escalating doses over time.^31^ In line with other studies, they noticed that IL-6, as well as colony stimulating factors (granulate-macrophage CSF [GM-CSF] and granulocyte CSF [G-CSF]), was produced more by IL-1β induction than TNF.^31^ Moreover, similar to our findings, TNF was not generated to any great extent following IL-1β stimulation.^31^ This clearly corroborates other studies showing that IL-1β seems to be the main inflammatory stimuli for astrocytes. Further, Lee and coworkers studied human fetal (16–24 weeks of gestation) primary cultures of astrocytes exposed to IL-1β and TNF (200 U/mL each), for up to 72 h. Only IL-1β stimulation led to increases in protein and mRNA over time for TNF, IL-1β, and IL-6.^32^ Interestingly, the authors noted a decrease in TNF production with IL-1β stimulation over time, indicating that it might not be continuously increasing over time like some of the other cytokines, which was also seen by Chung and Benveniste looking at rat astrocyte culture supernatants.^33^ This is similar to our results in which TNF induction did not induce as robust a response as IL-1β over time and could perhaps be explained by a relative downregulation of TNF activity over time. Similarly, studies show that *in vitro* lipopolysaccharide (LPS) stimulated astrocytes produced much less TNF mRNA than IL-1β, in comparison with microglia, which produced high amounts of both.^34,35^ These results appear to corroborate our findings and are in line with those of Liddelow and coworkers,^16^ in that TNF seems to be generated primarily by activated microglia and to a lesser extent by astrocytes, which could explain the TNF secretome results.

We have previously shown that IL-1 receptor 1 (IL-1R1) mRNA was more expressed in astrocyte cultures than in neuronal cultures.^17^ Similarly, we have now observed higher levels of IL-1, IL-6, and TNF receptor mRNA in astrocyte cultures than in neuronal cultures, whereas IL-4 and IL-10 receptor mRNA was relatively more highly expressed in neurons (Fig. 2). Although this can partly explain some of the varying degrees of downstream cytokine production in the secretome, the situation is probably more complex, with important pathways between different cytokines that are currently unexplored also having an effect. Tada and coworkers derived astrocyte cultures taken from the human brain after surgery, and looked at IL-1, TNF, and IL-6 receptors, and noted, as we did, that both IL-1 and TNF receptor mRNA was more expressed than IL-6.^36^ This is supported by Flynn and colleagues, who confirmed the role of TNF receptors on cultured astrocytes.^37^ A limitation is that previous studies have not investigated receptor upregulation for all of the cytokines used in this study, but the available literature appears to support IL-1β and TNF receptors being more upregulated than IL-4, IL-6, and IL-10, which corroborates the secretome results.

However, whereas the majority of groups stimulate astrocyte cultures with pro-inflammatory cytokines, there are groups that have studied the effect of added “anti-inflammatory” IL-4 and IL-10.^38–40^ Those three studies all use rat-derived astrocytes and add pro-inflammatory stimuli initially in order to see a subsequent anti-inflammatory response, so they are difficult to compare with our current study. Human astrocytes have been shown to present several different properties compared with rodent astrocytes. Human astrocytes are, for example, three times larger and have 10 times more processes than those of rodents.^41,42^ Additionally, rodent and human astrocytes differ in gene expression, with only about half of the genes most highly expressed in mouse astrocytes also expressed in human astrocytes, and only about one third of the genes most enriched in human astrocytes also expressed in the mouse.^43^ To our knowledge, there are no studies stimulating human iPSC-derived astrocytes with IL-4 and IL-10 in the ranges seen following TBI. Interestingly, both IL-4 and IL-10 stimulation lead to the production of similar cytokines, including more anti-inflammatory VEGF-A, as well as cytokines related to neutrophil recruitment: MMP9, M-CSF, and IL-8.

In experimental TBI, astrocytes have been shown to produce IL-6, which improves wound healing,^44^ but may also produce cytokines that further damage the blood–brain barrier and extracellular matrix.^45^ Similar properties have been attributed to the “glial scar” which is formed in the CNS following injury,^46^ driven in part by astrocyte-produced interferon (IFN)-γ.^47^ It is likely that the delicate interplay among all cells in the CNS plays an important role for astrocyte function and activity in the injured CNS. Liddelow and coworkers injected mice with LPS and noted that animals without microglia (CSF 1 receptor [CSF1R]^-/-^) did not generate A1 reactive astrocytes, concluding that microglial activation is necessary *in vivo* to produce A1 astrocytes.^16^ The necessity of providing microglia to generate an adequate astrocyte response is supported by previous *in vitro* work as well.^32^ In contrast, astrocytes co-cultured with microglia, as well as astrocyte culture medium transferred to microglial cultures, have been shown to downregulate microglial IL-12 production.^48^ This clearly describes a delicate interplay between these two cell types and that they both may interact and affect the downstream inflammatory response. A review has addressed this in the context of TBI as well,^49^ in which both the potentially harmful as well as the beneficial effects of astrocyte–microglia interactions are highlighted. In summary, although astrocytes may independently initiate an inflammatory response following stimuli, studies indicate that this is exacerbated by the presence of microglia, highlighting the need for further co-culture studies in which the exact inflammatory roles may be better elucidated.

When comparing the secretome response with that of neuronal cultures, TNF was produced at higher concentrations in response to stimulation of the astrocytes (primarily IL-1β, as can be seen in Table 2) which is presumably because of the difference in cytokine receptor expression.^17^ Looking at in *vivo* human samples, there were several cytokines with similar temporal trends, albeit at much lower levels, which is probably because of the minimalistic approach with only single cytokines added in the *in vitro* experiments. Noteworthy were high levels of IL-8 and G-CSF in the astrocyte cultures as compared with neuronal cultures, which could indicate that astrocytes play a greater role in neutrophil migration across the blood–brain barrier.^50,51^ Further, TNF production was relatively increased compared with neuronal cultures, being even higher than in *in vivo* concentrations, something that could be because the *in vivo* human studies missed the initial peak of TNF, which is thought to occur in the first 24 h following TBI.^7,52^

Our findings are significant for human TBI research, as we show that astrocytes in isolation (with no other cell types) are immunologically activated by the cytokine milieu seen in the aftermath of human TBI. Presumably, in combination with several pro-inflammatory cytokines and with microglia, this response will be escalated. This highlights the opportunity for anti-inflammatory drugs as a therapeutic option in the early aftermath of TBI.

### Limitations

The concentrations of cytokines and chemokines within our cell cultures are variable and we are reliant on the induction of cytokines being sufficiently large to reach statistical significance in multivariate ANOVA. We sought to screen a large number of cytokines and chemokines of interest from our previous studies in human TBI; however, it is possible that the more subtle effects of the added cytokines were not identified.

For the secretome analysis, noting that these experiments are both very expensive (each assay kit costs £3,200 and five kits were needed for the current results) and technically demanding (astrocytes take >100 days of culture to differentiate from human iPSCs), we have rationalized the samples used. Rather than using two lines (from different people), we used two independent neural inductions from the same (isogenic) line, as we reasoned that this would allow us to minimize inter-patient variability while increasing the power by conducting a second experimental block.

Further, cytokines are known to act in concert, and although we have sought a highly reductionist model in this study employing cytokines individually, we accept that combinations of cytokines would better reflect the situation in the injured human brain. This has to be balanced against the large numbers of experimental permutations that would be needed if multiple cytokines at multiple combinations were to be used. We have taken the approach of using the most robustly interpretable experimental paradigm. Having demonstrated that cytokines such as IL-1β and TNF can reliably induce a cytokine and chemokine response in cultured human astrocytes, corroborated with mRNA expression of the cognate receptors, this provides a rationale for combinations of these specific cytokines to be investigated.

Although the use of highly enriched astrocyte cultures does not attempt to model the cellular interactions between both glia and neurons in the human brain, we regard the present study as a requisite starting point demonstrating individual responses of astrocytes. This can then lead to formal co-culture experiments to enable understanding of the influence of heterotypic cellular interactions on secondary cytokine responses.

## Conclusion

Here we have shown that human iPSC-derived astrocytes exposed to cytokine concentrations reflecting those in severe TBI generate an increased downstream cytokine production, especially when exposed to IL-1β. This complements our previous work on neuronal cultures in which only IL-1β produced a few downstream cytokines. We have shown that these differences can be rationalized by different cell type-specific cytokine receptor expression. The escalating concentrations of cytokines did not significantly affect cellular viability. More work is needed to better understand how different cells in the CNS respond to the neuroinflammatory milieu after TBI, alone and in combination. Our model allows initial dissection of the response and contribution of astrocytes to neurotrauma in a clinically relevant model.

## Funding Information

E.P.T. is supported by post-doctoral scholarships from the Swedish Society for Medical Research and Swedish Society of Medicine (grant no. SLS-587221). K.L.H.C. is supported by the National Institute for Health Research Biomedical Research Centre, Cambridge (Neuroscience Theme; Brain Injury and Repair Theme). P.J.H. is supported by a Research Professorship from the National Institute for Health Research (NIHR), the NIHR Cambridge Biomedical Research Centre, the NIHR Global Health Research Group on Neurotrauma a European Union Seventh Framework Program grant (Collaborative European NeuroTrauma Effectiveness Research in TBI [CENTER-TBI]; grant no. 602150), and the Royal College of Surgeons of England. R.P. is supported by an Medical Research Council (MRC)/Motor Neuron Disease Association (MNDA) Lady Edith Wolfson Senior Clinical Fellowship (grant no. MR/S006591/1). We also acknowledge support from the National Institute for Health Research University College London Hospital Biomedical Research Centre. A.H. is supported by the Medical Research Council/Royal College of Surgeons of England Clinical Research Training Fellowship, Royal College of Surgeons of England Pump Priming Grant, and the National Institute for Health Research Biomedical Research Centre, Cambridge. The Luminex 200 analyzer was purchased with MRC funding (grant no. G0600986 ID79068). We acknowledge the Dementias Platform UK (DPUK)/MRC platform for provision of the Opera Phenix for high-throughput iPSC analysis. TT is supported by the National Institute of Health Research. AA is supported by a Newton Fellowship from the Academy of Medical Sciences. The funding bodies did not participate in the design of the study; collection, analysis, and interpretation of data; or writing of the article.

## Supplementary Material

Supplemental data

Supplemental data

Supplemental data

Supplemental data

Supplemental data
