# Supplementary material for: Delineating Astrocytic Cytokine Responses in a Human Stem Cell Model of Neural Trauma
Source: J Neurotrauma. 2019 Dec 11;37(1):93–105. doi: 10.1089/neu.2019.6480 (PMC6921298; doi:10.1089/neu.2019.6480)
Supplement: Supplemental data [file Supp_TableS1-S2.pdf]

Supplementary Table S1. Proteins Where RNA Levels Were Sequenced

Cytokine receptors	
IL1R1	IL-1 receptor 1
IL1RAP	IL-1 receptor accessory protein
IL4R	IL-4 receptor
IL6R	IL-6 receptor
IL6ST	IL-6 signal transducer
IL10RA	IL-10 receptor A
IL10RB	IL-10 receptor B
TNFRSF1A (also known as TNFR1)	TNF receptor superfamily 1A
TNFRSF1B (also known as TNFBR)	TNF receptor superfamily 1B

Supplementary Table S2. Patient Demographics

Patient	*Age*	*Sex*	*Injury mechanism*	*Post-injury GCS*	*Type of injury*
Patients used to determine temporal profile of cytokines					
1	28	Male	Fall down stairs	E1 V1 M2 = 4	Diffuse
2	28	Male	Road traffic accident	E2 V1 M5 = 8	Diffuse
3	26	Male	Road traffic accident	E2 V1 M4 = 7	Diffuse
4	35	Male	Assault	E1 V1 M4 = 6	Focal mass lesion
5	52	Female	Road traffic accident	E1 V1 M5 = 7	Diffuse
6	18	Female	Road traffic accident	E1 V1 M1 = 3	Diffuse
7	44	Female	Road traffic accident	E1 V1 M1 = 3	Diffuse
8	25	Male	Road traffic accident	E1 V1 M1 = 3	Diffuse
9	27	Female	Road traffic accident	E1 V1 M1 = 3	Diffuse
10	25	Female	Road traffic accident	E1 V1 M2 = 4	Diffuse
11	58	Female	Road traffic accident	E1 V1 M1 = 3	Diffuse
12	61	Female	Road traffic accident	E1 V1 M1 = 3	Diffuse
Patients used to assess cytokine levels					
1	44	Female	Road traffic accident	E1 V1 M1 = 3	Diffuse
2	25	Male	Road traffic accident	E1 V1 M1 = 3	Diffuse
3	27	Female	Road traffic accident	E1 V1 M1 = 3	Diffuse
4	25	Female	Road traffic accident	E1 V1 M2 = 4	Diffuse
5	58	Female	Road traffic accident	E1 V1 M1 = 3	Focal mass lesion
6	61	Female	Road traffic accident	E1 V1 M1 = 3	Focal mass lesion
7	49	Male	Road traffic accident	E1 V2 M5 = 8	Focal mass lesion
8	60	Male	Road traffic accident	E1 V1 M5 = 7	Diffuse
9	30	Male	Fall	E1 V2 M2 = 5	Focal mass lesion
10	39	Female	Road traffic accident	E1 V1 M1 = 3	Diffuse

diffuse injury, Marshall CT classification grades II–IV; focal mass lesion, Marshall CT classification grade VI; GCS, Glasgow Coma Scale; E, eye component; V, verbal component; M, motor component.
