# Supplementary material for: Delineating Astrocytic Cytokine Responses in a Human Stem Cell Model of Neural Trauma
Source: J Neurotrauma. 2019 Dec 11;37(1):93–105. doi: 10.1089/neu.2019.6480 (PMC6921298; doi:10.1089/neu.2019.6480)
Supplement: Supplemental data [file Supp_FigS1.pdf]

SUPPLEMENTARY FIG. S1. Bar graph illustrating analysis of variance (ANOVA) models of downstream cytokine stimulation with significant results. ANOVA models displaying models of testing cytokines significant over time, concentration vs. time × concentration over the time points 24 h, 48 h and 72 h for the different concentrations of stimulating cytokines. In the lower section of each figure is an ANOVA table of significance (p value) between different groups.

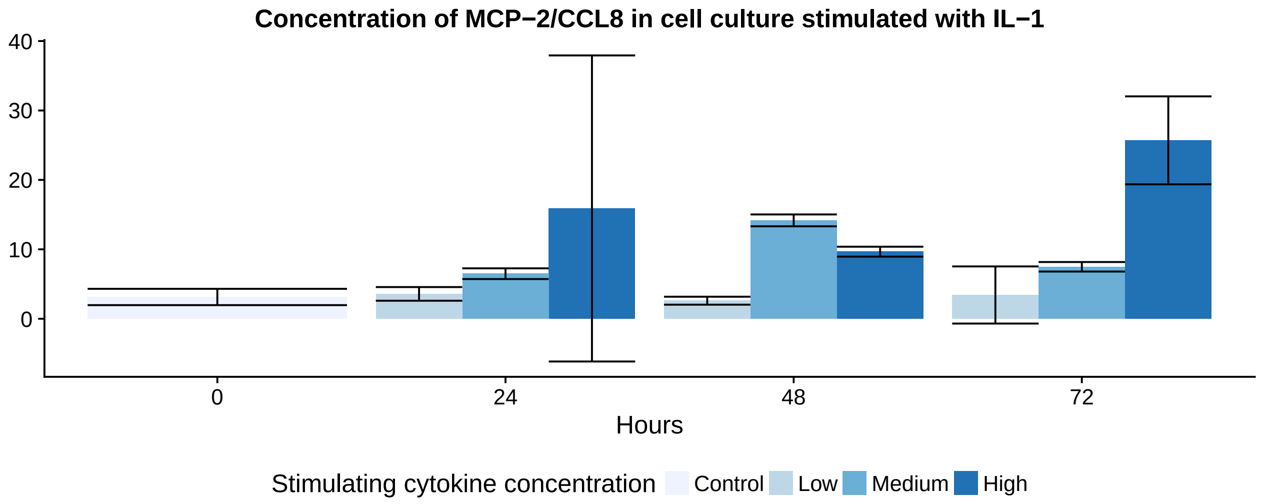

	Df	Sum Sq	Mean Sq	F value	Pr(>F)
Time	1	224.5627	224.5627	5.608165	0.0280643
Concentration	1	468.2271	468.2271	11.693371	0.0027160
Time:Concentration	1	177.7141	177.7141	4.438182	0.0479700
Residuals	20	800.8419	40.0421	NA	NA

	0 0	24 1	24 2	24 3	48 1	48 2	48 3	72 1	72 2
24 1	1.0000000	NA	NA	NA	NA	NA	NA	NA	NA
24 2	1.0000000	1.0000000	NA	NA	NA	NA	NA	NA	NA
24 3	1.0000000	1.0000000	1.000000	NA	NA	NA	NA	NA	NA
48 1	1.0000000	1.0000000	1.000000	1	NA	NA	NA	NA	NA
48 2	1.0000000	1.0000000	1.000000	1	1.0000000	NA	NA	NA	NA
48 3	1.0000000	1.0000000	1.000000	1	1.0000000	1	NA	NA	NA
72 1	1.0000000	1.0000000	1.000000	1	1.0000000	1	1	NA	NA
72 2	1.0000000	1.0000000	1.000000	1	1.0000000	1	1	1.0000000	NA
72 3	0.0275072	0.1537133	0.385785	1	0.1128605	1	1	0.1462983	0.5278666

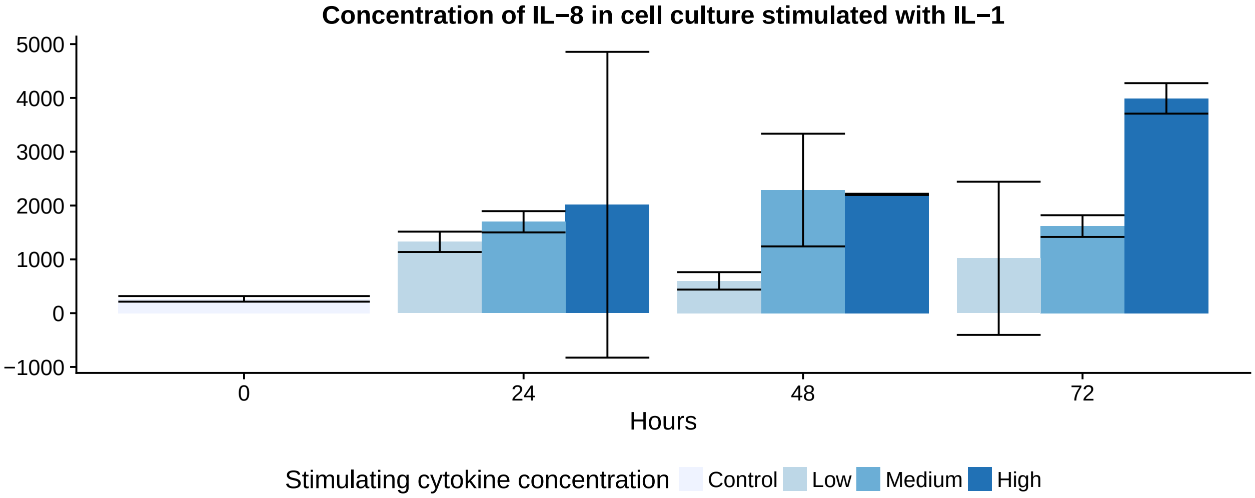

	Df	Sum Sq	Mean Sq	F value	Pr(>F)
Time	1	10272194	10272194	13.995273	0.0012880
Concentration	1	10768441	10768441	14.671380	0.0010458
Time:Concentration	1	2260452	2260452	3.079736	0.0945816
Residuals	20	14679519	733976	NA	NA

	0 0	24 1	24 2	24 3	48 1	48 2	48 3	72 1	72 2
24 1	1.0000000	NA	NA	NA	NA	NA	NA	NA	NA
24 2	1.0000000	1.0000000	NA	NA	NA	NA	NA	NA	NA
24 3	1.0000000	1.0000000	1	NA	NA	NA	NA	NA	NA
48 1	1.0000000	1.0000000	1	1	NA	NA	NA	NA	NA
48 2	0.7164578	1.0000000	1	1	1.0000000	NA	NA	NA	NA
48 3	0.8870702	1.0000000	1	1	1.0000000	1	NA	NA	NA
72 1	1.0000000	1.0000000	1	1	1.0000000	1	1	NA	NA
72 2	1.0000000	1.0000000	1	1	1.0000000	1	1	1.0000000	NA
72 3	0.0079803	0.4746242	1	1	0.0963698	1	1	0.2419956	0.8974713

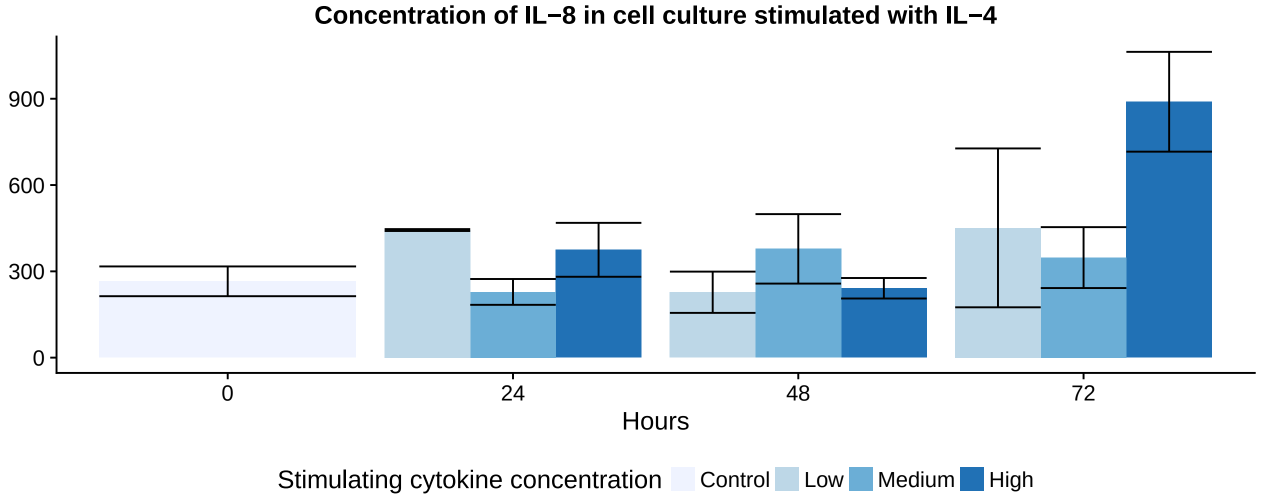

	Df	Sum Sq	Mean Sq	F value	Pr(>F)
Time	1	204571.55	204571.55	7.5280776	0.0125177
Concentration	1	11750.44	11750.44	0.4324073	0.5183073
Time:Concentration	1	147009.20	147009.20	5.4098266	0.0306531
Residuals	20	543489.49	27174.47	NA	NA

	0 0	24 1	24 2	24 3	48 1	48 2	48 3	72 1	72 2
24 1	1.0000000	NA	NA	NA	NA	NA	NA	NA	NA
24 2	1.0000000	1.0000000	NA	NA	NA	NA	NA	NA	NA
24 3	1.0000000	1.0000000	1.0000000	NA	NA	NA	NA	NA	NA
48 1	1.0000000	1.0000000	1.0000000	1.0000000	NA	NA	NA	NA	NA
48 2	1.0000000	1.0000000	1.0000000	1.0000000	1.0000000	NA	NA	NA	NA
48 3	1.0000000	1.0000000	1.0000000	1.0000000	1.0000000	1.0000000	NA	NA	NA
72 1	1.0000000	1.0000000	1.0000000	1.0000000	1.0000000	1.0000000	1.0000000	NA	NA
72 2	1.0000000	1.0000000	1.0000000	1.0000000	1.0000000	1.0000000	1.0000000	1.0000000	NA
72 3	0.0002438	0.0453614	0.0011704	0.0133605	0.0011489	0.0141647	0.0014329	0.0520384	0.0083638

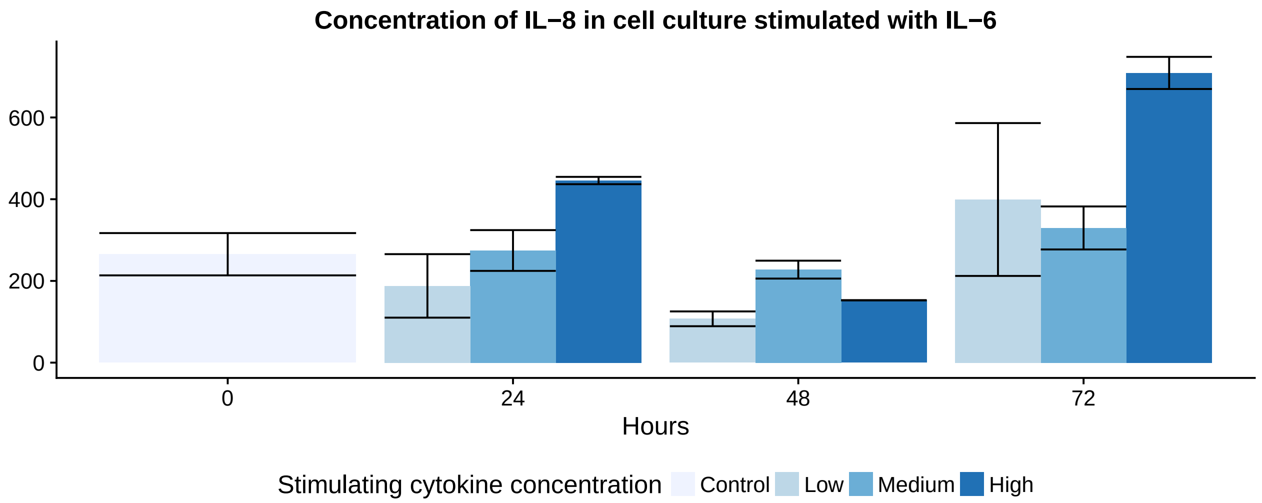

	Df	Sum Sq	Mean Sq	F value	Pr(>F)
Time	1	75536.00	75536.00	3.613127	0.0718350
Concentration	1	30739.86	30739.86	1.470385	0.2394146
Time:Concentration	1	97914.45	97914.45	4.683559	0.0427291
Residuals	20	418119.88	20905.99	NA	NA

	0 0	24 1	24 2	24 3	48 1	48 2	48 3	72 1	72 2
24 1	1.0000000	NA	NA	NA	NA	NA	NA	NA	NA
24 2	1.0000000	1.0000000	NA	NA	NA	NA	NA	NA	NA
24 3	0.2292107	0.0762158	0.9990091	NA	NA	NA	NA	NA	NA
48 1	0.5171916	1.0000000	1.0000000	0.0075360	NA	NA	NA	NA	NA
48 2	1.0000000	1.0000000	1.0000000	0.2494568	1.0000000	NA	NA	NA	NA
48 3	1.0000000	1.0000000	1.0000000	0.0272401	1.0000000	1.0000000	NA	NA	NA
72 1	1.0000000	0.3039527	1.0000000	1.0000000	0.0280784	0.9877810	0.1064736	NA	NA
72 2	1.0000000	1.0000000	1.0000000	1.0000000	0.2191947	1.0000000	0.8439958	1.0000000	NA
72 3	0.0000490	0.0000796	0.0006057	0.0648410	0.0000146	0.0001968	0.0000371	0.0168475	0.0024862

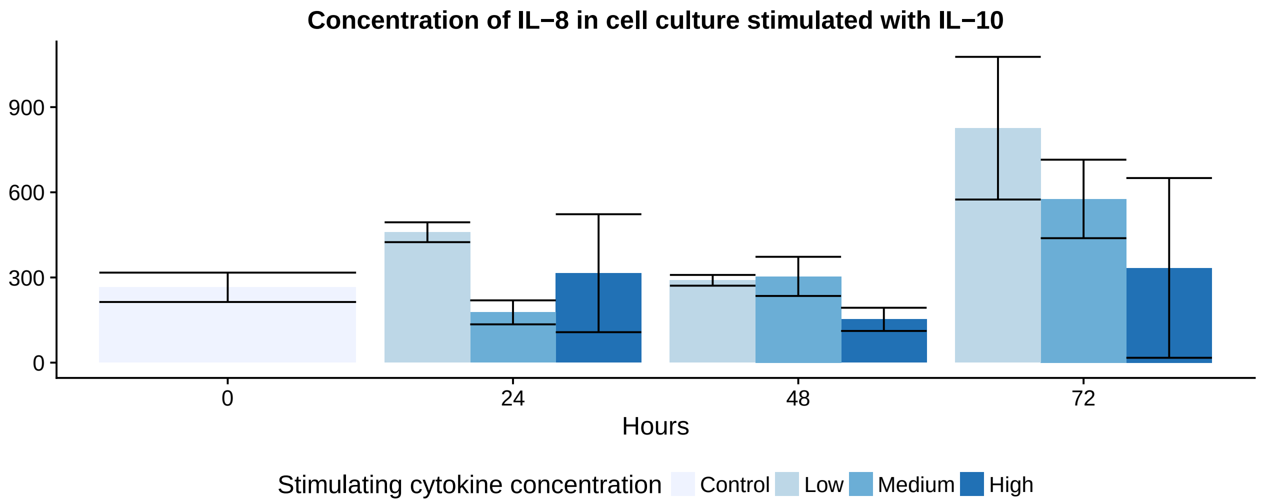

	Df	Sum Sq	Mean Sq	F value	Pr(>F)
Time	1	227892.02	227892.02	8.828290	0.0075485
Concentration	1	223372.45	223372.45	8.653207	0.0080669
Time:Concentration	1	55669.61	55669.61	2.156580	0.1575136
Residuals	20	516276.70	25813.84	NA	NA

	0 0	24 1	24 2	24 3	48 1	48 2	48 3	72 1	72 2
24 1	1.0000000	NA	NA	NA	NA	NA	NA	NA	NA
24 2	1.0000000	1.0000000	NA	NA	NA	NA	NA	NA	NA
24 3	1.0000000	1.0000000	1.0000000	NA	NA	NA	NA	NA	NA
48 1	1.0000000	1.0000000	1.0000000	1.0000000	NA	NA	NA	NA	NA
48 2	1.0000000	1.0000000	1.0000000	1.0000000	1.0000000	NA	NA	NA	NA
48 3	1.0000000	1.0000000	1.0000000	1.0000000	1.0000000	1.0000000	NA	NA	NA
72 1	0.0066436	0.7055583	0.0112387	0.0824882	0.0570009	0.0698676	0.0079635	NA	NA
72 2	0.5673397	1.0000000	0.4330682	1.0000000	1.0000000	1.0000000	0.2997869	1.0000000	NA
72 3	1.0000000	1.0000000	1.0000000	1.0000000	1.0000000	1.0000000	1.0000000	0.1086413	1

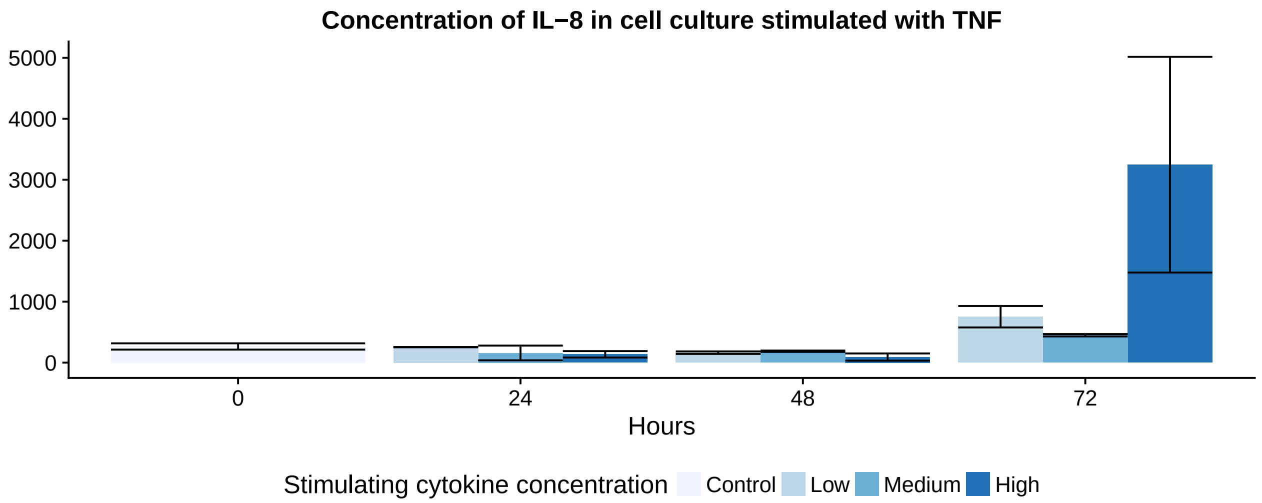

	Df	Sum Sq	Mean Sq	F value	Pr(>F)
Time	1	3927133.86	3927133.86	8.1658854	0.0097312
Concentration	1	68829.02	68829.02	0.1431196	0.7091849
Time:Concentration	1	6501288.31	6501288.31	13.5184532	0.0014961
Residuals	20	9618390.82	480919.54	NA	NA

	0 0	24 1	24 2	24 3	48 1	48 2	48 3	72 1	72 2
24 1	1.0000000	NA	NA	NA	NA	NA	NA	NA	NA
24 2	1.0000000	1.000000	NA	NA	NA	NA	NA	NA	NA
24 3	1.0000000	1.000000	1.0000000	NA	NA	NA	NA	NA	NA
48 1	1.0000000	1.000000	1.0000000	1.0000000	NA	NA	NA	NA	NA
48 2	1.0000000	1.000000	1.0000000	1.0000000	1.0000000	NA	NA	NA	NA
48 3	1.0000000	1.000000	1.0000000	1.0000000	1.0000000	1.0000000	NA	NA	NA
72 1	1.0000000	1.000000	1.0000000	1.0000000	1.0000000	1.0000000	1.0000000	NA	NA
72 2	1.0000000	1.000000	1.0000000	1.0000000	1.0000000	1.0000000	1.0000000	1.0000000	NA
72 3	0.0001048	0.000939	0.0006724	0.0006229	0.0006835	0.0007419	0.0005337	0.0058681	0.0018896

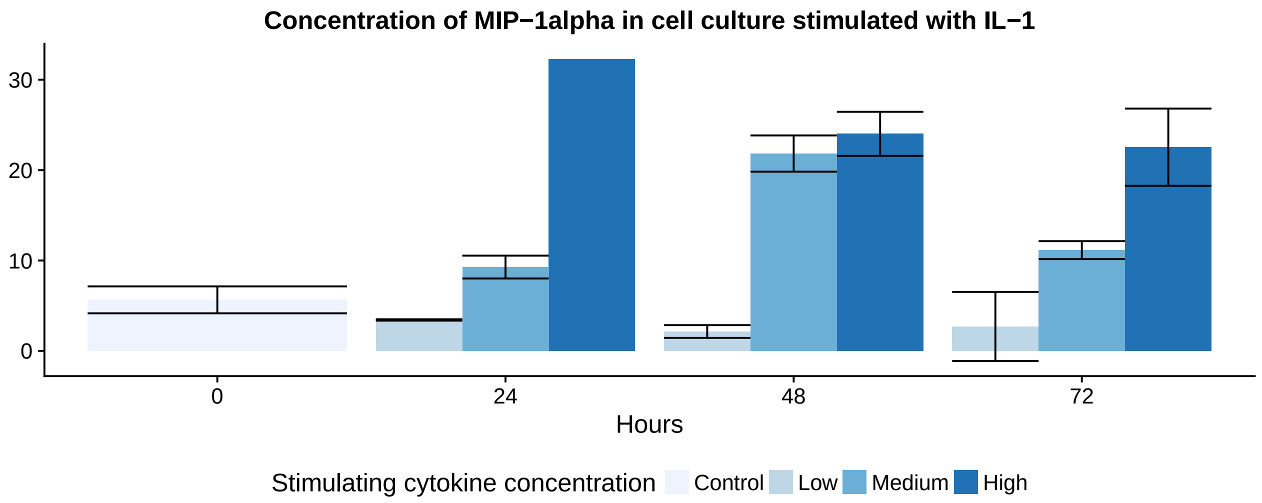

	Df	Sum Sq	Mean Sq	F value	Pr(>F)
Time	1	173.36656	173.36656	8.328650	0.0094665
Concentration	1	1292.91636	1292.91636	62.112598	0.0000002
Time:Concentration	1	73.39504	73.39504	3.525949	0.0758531
Residuals	19	395.49804	20.81569	NA	NA

	0 0	24 1	24 2	24 3	48 1	48 2	48 3	72 1	72 2
24 1	NA	NA	NA	NA	NA	NA	NA	NA	NA
24 2	NA	NA	NA	NA	NA	NA	NA	NA	NA
24 3	NA	NA	NA	NA	NA	NA	NA	NA	NA
48 1	NA	NA	NA	NA	NA	NA	NA	NA	NA
48 2	NA	NA	NA	NA	NA	NA	NA	NA	NA
48 3	NA	NA	NA	NA	NA	NA	NA	NA	NA
72 1	NA	NA	NA	NA	NA	NA	NA	NA	NA
72 2	NA	NA	NA	NA	NA	NA	NA	NA	NA
72 3	NA	NA	NA	NA	NA	NA	NA	NA	NA

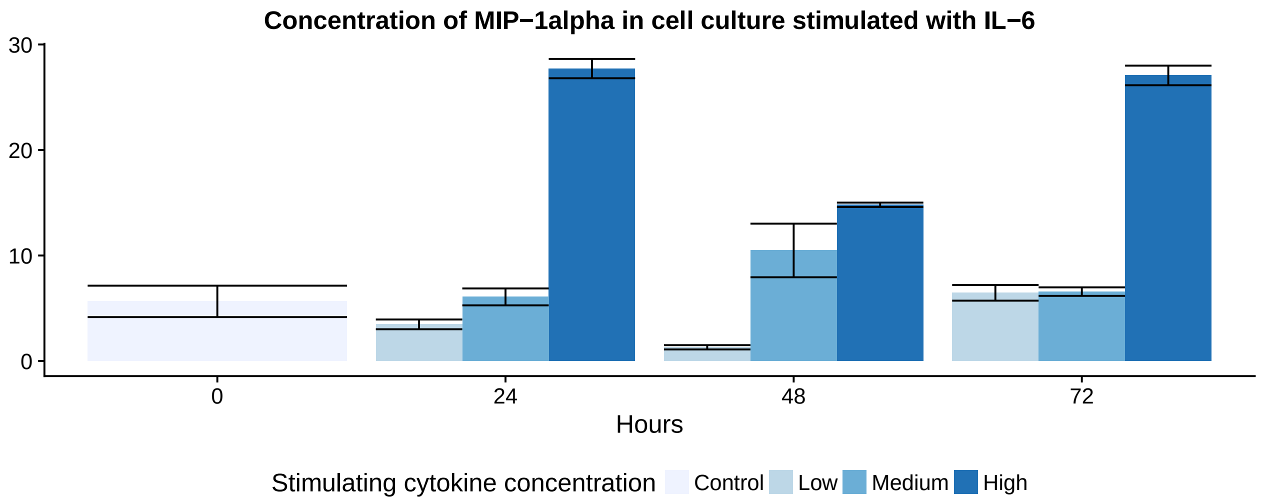

	Df	Sum Sq	Mean Sq	F value	Pr(>F)
Time	1	115.1187	115.11868	4.429943	0.0481583
Concentration	1	942.6741	942.67413	36.275547	0.0000069
Time:Concentration	1	129.1784	129.17835	4.970981	0.0373996
Residuals	20	519.7298	25.98649	NA	NA

	0 0	24 1	24 2	24 3	48 1	48 2	48 3	72 1	72 2
24 1	1.0000000	NA	NA	NA	NA	NA	NA	NA	NA
24 2	1.0000000	1.0000000	NA	NA	NA	NA	NA	NA	NA
24 3	0.0000000	0.0000000	0.0000000	NA	NA	NA	NA	NA	NA
48 1	0.0293643	1.0000000	0.0705233	0.0e+00	NA	NA	NA	NA	NA
48 2	0.0117835	0.0023237	0.1300165	1.0e-07	0.0001271	NA	NA	NA	NA
48 3	0.0000120	0.0000106	0.0002248	2.1e-06	0.0000012	0.1467528	NA	NA	NA
72 1	1.0000000	1.0000000	1.0000000	0.0e+00	0.0383515	0.2415238	0.0003683	NA	NA
72 2	1.0000000	1.0000000	1.0000000	0.0e+00	0.0317796	0.2929402	0.0004306	1	NA
72 3	0.0000000	0.0000000	0.0000000	1.0e+00	0.0000000	0.0000001	0.0000040	0	0

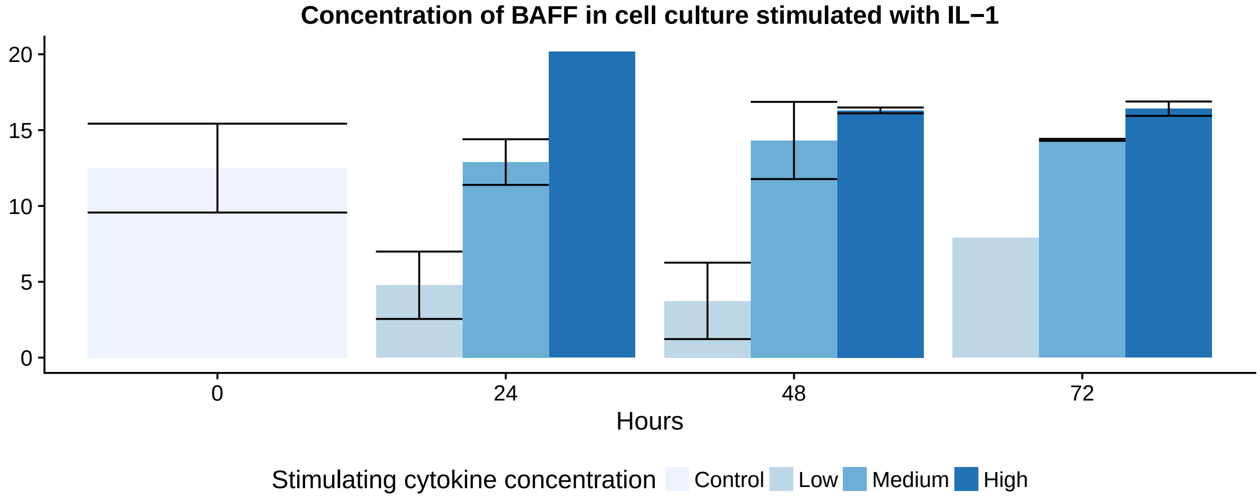

	Df	Sum Sq	Mean Sq	F value	Pr(>F)
Time	1	9.225729	9.225729	0.7779055	0.3926742
Concentration	1	225.414591	225.414591	19.0067647	0.0006537
Time:Concentration	1	51.898755	51.898755	4.3760585	0.0551527
Residuals	14	166.035847	11.859703	NA	NA

	0 0	24 1	24 2	24 3	48 1	48 2	48 3	72 1	72 2
24 1	NA	NA	NA	NA	NA	NA	NA	NA	NA
24 2	NA	NA	NA	NA	NA	NA	NA	NA	NA
24 3	NA	NA	NA	NA	NA	NA	NA	NA	NA
48 1	NA	NA	NA	NA	NA	NA	NA	NA	NA
48 2	NA	NA	NA	NA	NA	NA	NA	NA	NA
48 3	NA	NA	NA	NA	NA	NA	NA	NA	NA
72 1	NA	NA	NA	NA	NA	NA	NA	NA	NA
72 2	NA	NA	NA	NA	NA	NA	NA	NA	NA
72 3	NA	NA	NA	NA	NA	NA	NA	NA	NA

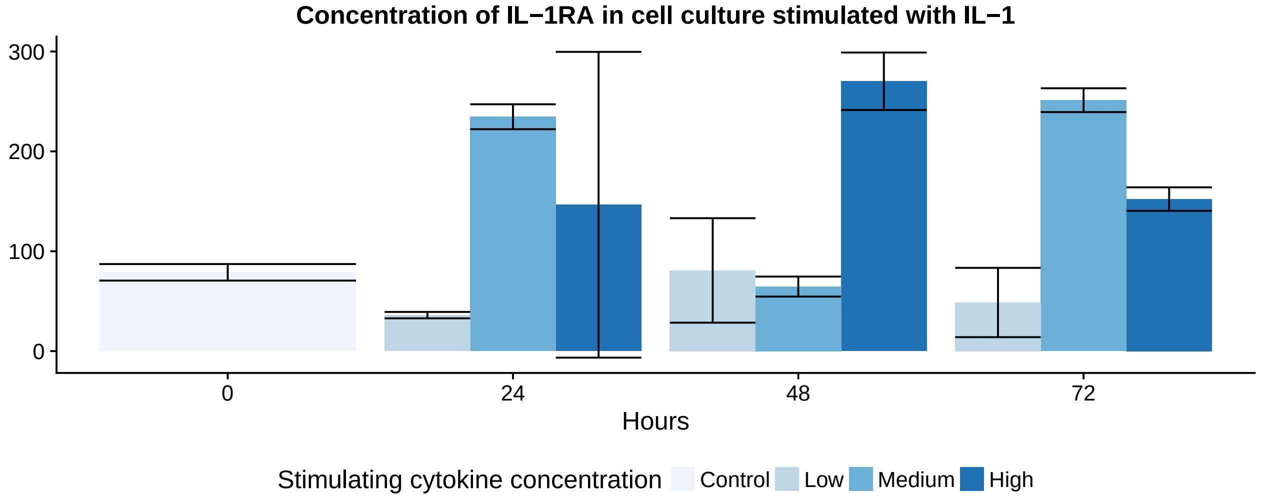

	Df	Sum Sq	Mean Sq	F value	Pr(>F)
Time	1	11738.007	11738.007	2.0176429	0.1716832
Concentration	1	51559.818	51559.818	8.8626031	0.0077458
Time:Concentration	1	4635.617	4635.617	0.7968149	0.3832140
Residuals	19	110535.983	5817.683	NA	NA

	0 0	24 1	24 2	24 3	48 1	48 2	48 3	72 1	72 2
24 1	1.0000000	NA	NA	NA	NA	NA	NA	NA	NA
24 2	0.0761019	0.0464503	NA	NA	NA	NA	NA	NA	NA
24 3	1.0000000	1.0000000	1.0000000	NA	NA	NA	NA	NA	NA
48 1	1.0000000	1.0000000	0.2808968	1.0000000	NA	NA	NA	NA	NA
48 2	1.0000000	1.0000000	0.1455746	1.0000000	1.0000000	NA	NA	NA	NA
48 3	0.0145592	0.0118332	1.0000000	0.9597493	0.0669499	0.0353505	NA	NA	NA
72 1	1.0000000	1.0000000	0.0766925	1.0000000	1.0000000	1.0000000	0.0190929	NA	NA
72 2	0.0347583	0.0243190	1.0000000	1.0000000	0.1432018	0.0747038	1.0000000	0.0397564	NA
72 3	1.0000000	1.0000000	1.0000000	1.0000000	1.0000000	1.0000000	1.0000000	1.0000000	1

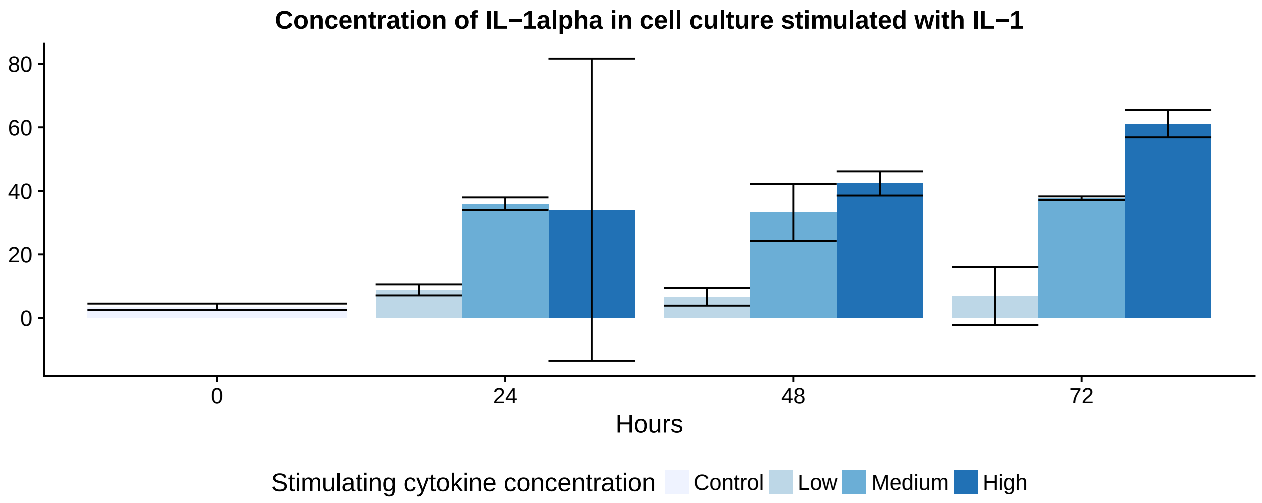

	Df	Sum Sq	Mean Sq	F value	Pr(>F)
Time	1	2782.377	2782.377	18.822484	0.0003189
Concentration	1	4441.065	4441.065	30.043332	0.0000230
Time:Concentration	1	904.535	904.535	6.119084	0.0224589
Residuals	20	2956.439	147.822	NA	NA

	0 0	24 1	24 2	24 3	48 1	48 2	48 3	72 1	72 2
24 1	1.0000000	NA	NA	NA	NA	NA	NA	NA	NA
24 2	0.4400445	1.0000000	NA	NA	NA	NA	NA	NA	NA
24 3	0.6236331	1.0000000	1	NA	NA	NA	NA	NA	NA
48 1	1.0000000	1.0000000	1	1	NA	NA	NA	NA	NA
48 2	0.7272581	1.0000000	1	1	1.0000000	NA	NA	NA	NA
48 3	0.1377560	1.0000000	1	1	0.8030434	1	NA	NA	NA
72 1	1.0000000	1.0000000	1	1	1.0000000	1	0.8406569	NA	NA
72 2	0.3221923	1.0000000	1	1	1.0000000	1	1.0000000	1.0000000	NA
72 3	0.0050209	0.0675833	1	1	0.0490997	1	1.0000000	0.0513986	1

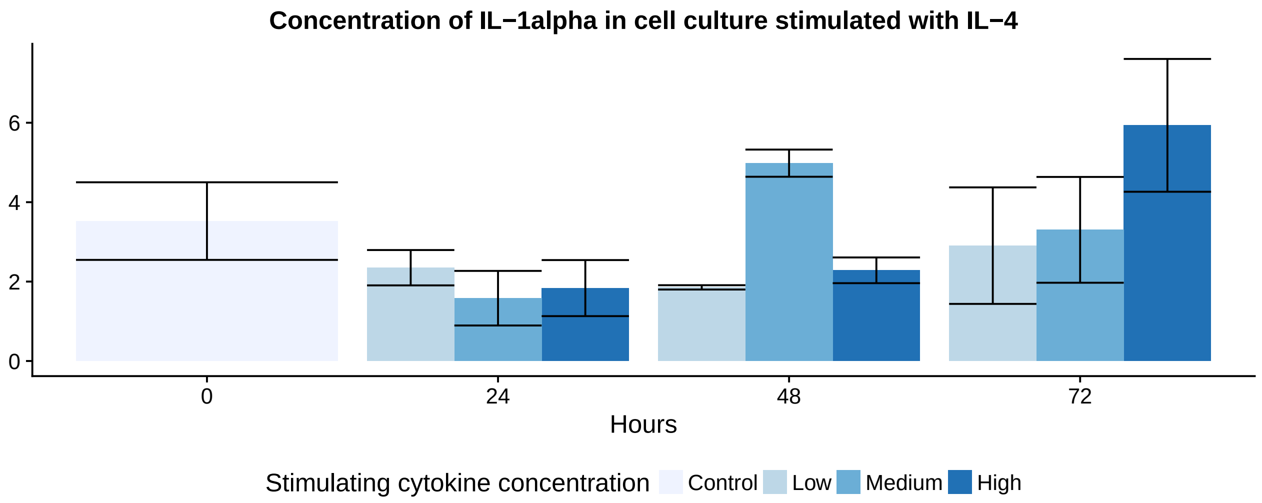

	Df	Sum Sq	Mean Sq	F value	Pr(>F)
Time	1	2.1702920	2.1702920	1.6263281	0.2168269
Concentration	1	0.6875831	0.6875831	0.5152467	0.4811776
Time:Concentration	1	21.0721518	21.0721518	15.7906090	0.0007481
Residuals	20	26.6894731	1.3344737	NA	NA

	0 0	24 1	24 2	24 3	48 1	48 2	48 3	72 1	72 2
24 1	1.0000000	NA	NA	NA	NA	NA	NA	NA	NA
24 2	1.0000000	1.0000000	NA	NA	NA	NA	NA	NA	NA
24 3	1.0000000	1.0000000	1.0000000	NA	NA	NA	NA	NA	NA
48 1	1.0000000	1.0000000	1.0000000	1.0000000	NA	NA	NA	NA	NA
48 2	1.0000000	0.7136490	0.1461093	0.2473228	0.2569927	NA	NA	NA	NA
48 3	1.0000000	1.0000000	1.0000000	1.0000000	1.0000000	0.6240327	NA	NA	NA
72 1	1.0000000	1.0000000	1.0000000	1.0000000	1.0000000	1.0000000	1.0000000	NA	NA
72 2	1.0000000	1.0000000	1.0000000	1.0000000	1.0000000	1.0000000	1.0000000	1.0000000	NA
72 3	0.3691581	0.0997028	0.0209961	0.0349385	0.0362687	1.0000000	0.0871222	0.3151903	0.71511

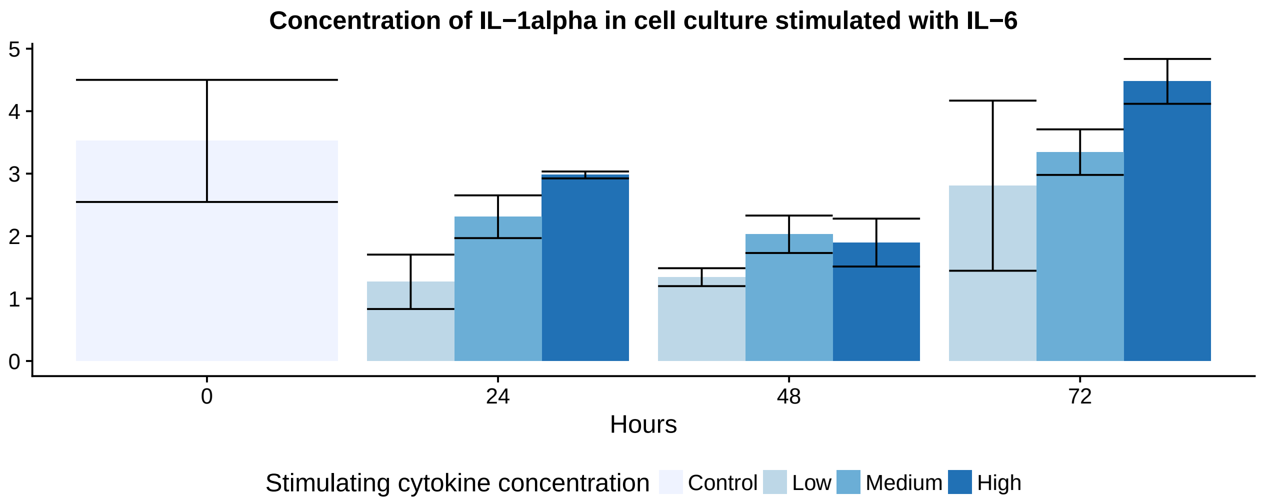

	Df	Sum Sq	Mean Sq	F value	Pr(>F)
Time	1	0.0417014	0.0417014	0.0451736	0.8338364
Concentration	1	0.0270300	0.0270300	0.0292806	0.8658521
Time:Concentration	1	10.5429882	10.5429882	11.4208301	0.0029790
Residuals	20	18.4627354	0.9231368	NA	NA

	0 0	24 1	24 2	24 3	48 1	48 2	48 3	72 1	72 2
24 1	0.0905413	NA	NA	NA	NA	NA	NA	NA	NA
24 2	1.0000000	1.0000000	NA	NA	NA	NA	NA	NA	NA
24 3	1.0000000	1.0000000	1.0000000	NA	NA	NA	NA	NA	NA
48 1	0.1159464	1.0000000	1.0000000	1	NA	NA	NA	NA	NA
48 2	1.0000000	1.0000000	1.0000000	1	1.0000000	NA	NA	NA	NA
48 3	0.7311266	1.0000000	1.0000000	1	1.0000000	1.0000000	NA	NA	NA
72 1	1.0000000	1.0000000	1.0000000	1	1.0000000	1.0000000	1.0000000	NA	NA
72 2	1.0000000	0.5853638	1.0000000	1	0.7155280	1.0000000	1.0000000	1	NA
72 3	1.0000000	0.0274359	0.4575401	1	0.0334093	0.2129419	0.1482301	1	1

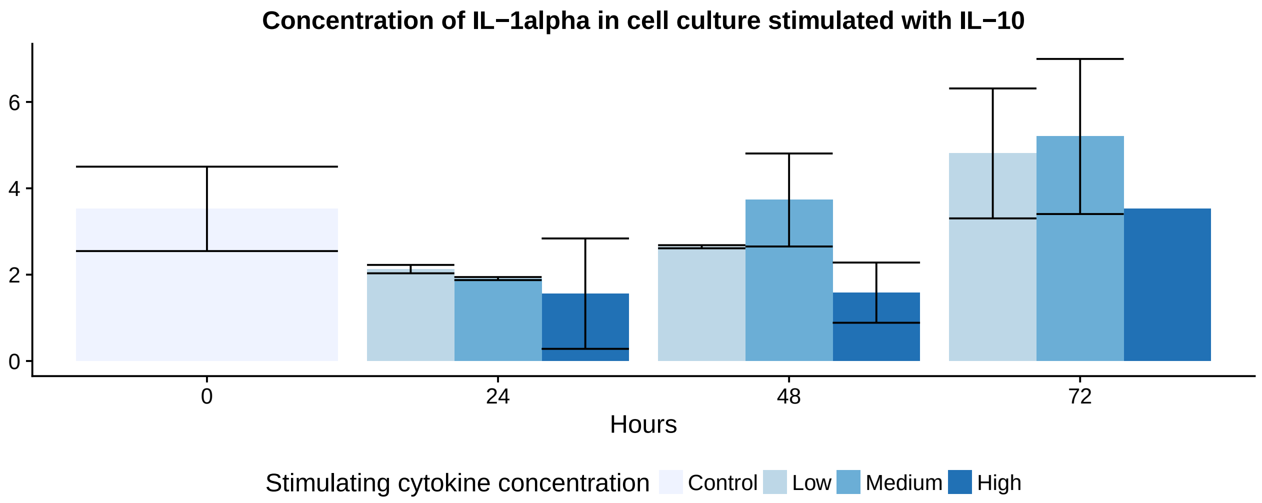

	Df	Sum Sq	Mean Sq	F value	Pr(>F)
Time	1	4.099480	4.099480	3.487270	0.0773482
Concentration	1	15.524657	15.524657	13.206230	0.0017662
Time:Concentration	1	2.777236	2.777236	2.362488	0.1407705
Residuals	19	22.335556	1.175556	NA	NA

	0 0	24 1	24 2	24 3	48 1	48 2	48 3	72 1	72 2
24 1	NA	NA	NA	NA	NA	NA	NA	NA	NA
24 2	NA	NA	NA	NA	NA	NA	NA	NA	NA
24 3	NA	NA	NA	NA	NA	NA	NA	NA	NA
48 1	NA	NA	NA	NA	NA	NA	NA	NA	NA
48 2	NA	NA	NA	NA	NA	NA	NA	NA	NA
48 3	NA	NA	NA	NA	NA	NA	NA	NA	NA
72 1	NA	NA	NA	NA	NA	NA	NA	NA	NA
72 2	NA	NA	NA	NA	NA	NA	NA	NA	NA
72 3	NA	NA	NA	NA	NA	NA	NA	NA	NA

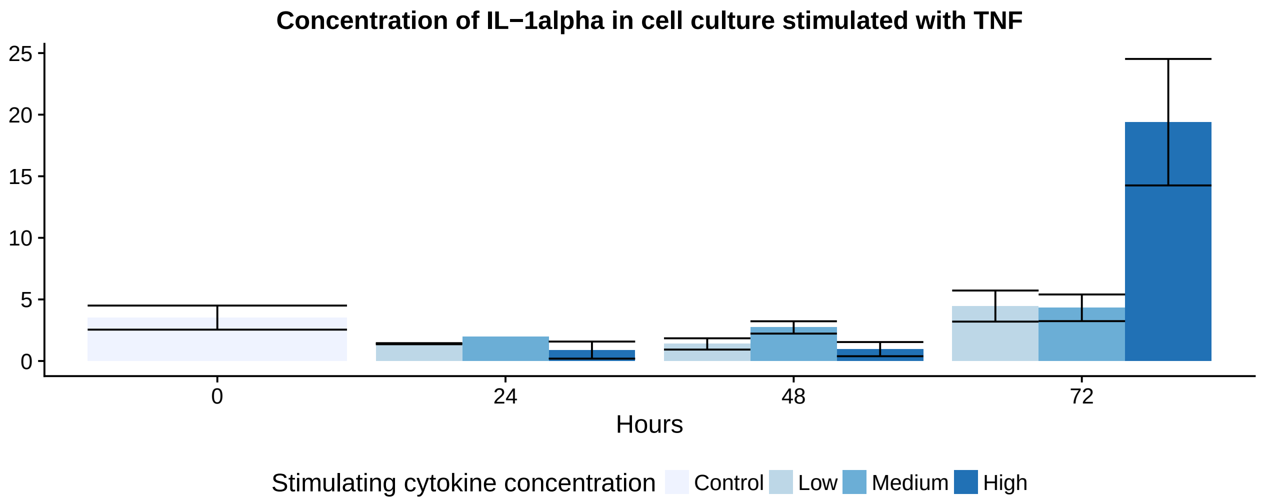

	Df	Sum Sq	Mean Sq	F value	Pr(>F)
Time	1	92.840639	92.840639	8.4111760	0.0091746
Concentration	1	0.357396	0.357396	0.0323794	0.8591028
Time:Concentration	1	279.985410	279.985410	25.3661175	0.0000733
Residuals	19	209.717659	11.037771	NA	NA

	0 0	24 1	24 2	24 3	48 1	48 2	48 3	72 1	72 2
24 1	NA	NA	NA	NA	NA	NA	NA	NA	NA
24 2	NA	NA	NA	NA	NA	NA	NA	NA	NA
24 3	NA	NA	NA	NA	NA	NA	NA	NA	NA
48 1	NA	NA	NA	NA	NA	NA	NA	NA	NA
48 2	NA	NA	NA	NA	NA	NA	NA	NA	NA
48 3	NA	NA	NA	NA	NA	NA	NA	NA	NA
72 1	NA	NA	NA	NA	NA	NA	NA	NA	NA
72 2	NA	NA	NA	NA	NA	NA	NA	NA	NA
72 3	NA	NA	NA	NA	NA	NA	NA	NA	NA

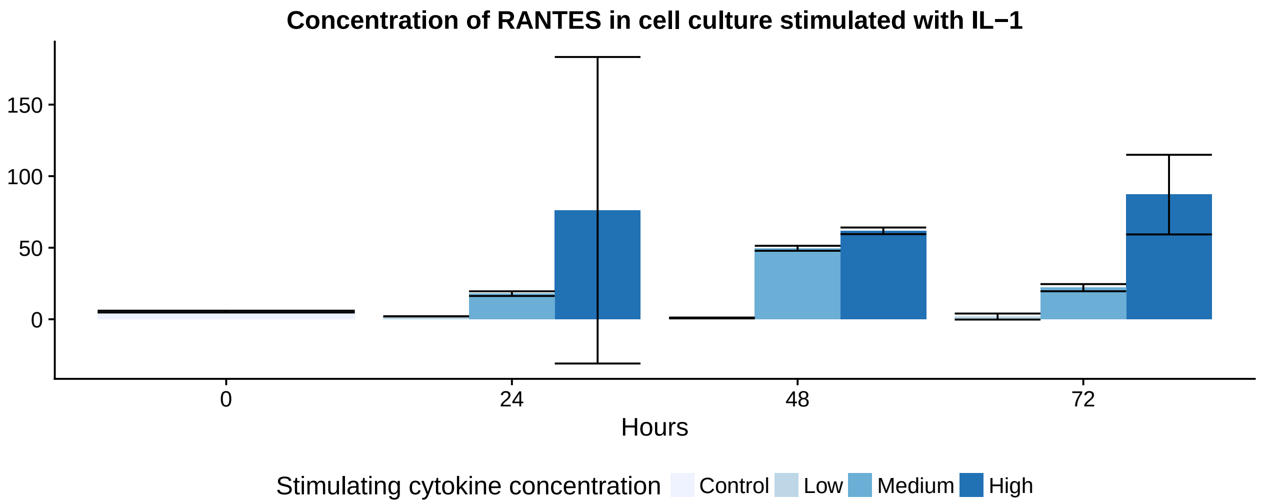

	Df	Sum Sq	Mean Sq	F value	Pr(>F)
Time	1	2554.072	2554.0723	3.295317	0.0852957
Concentration	1	14717.602	14717.6019	18.988956	0.0003389
Time:Concentration	1	2113.097	2113.0967	2.726361	0.1151361
Residuals	19	14726.162	775.0611	NA	NA

	0 0	24 1	24 2	24 3	48 1	48 2	48 3	72 1	72 2
24 1	1.0000000	NA	NA	NA	NA	NA	NA	NA	NA
24 2	1.0000000	1.0000000	NA	NA	NA	NA	NA	NA	NA
24 3	0.7368434	1.0000000	1	NA	NA	NA	NA	NA	NA
48 1	1.0000000	1.0000000	1	1	NA	NA	NA	NA	NA
48 2	1.0000000	1.0000000	1	1	1.000000	NA	NA	NA	NA
48 3	1.0000000	1.0000000	1	1	1.000000	1	NA	NA	NA
72 1	1.0000000	1.0000000	1	1	1.000000	1	1	NA	NA
72 2	1.0000000	1.0000000	1	1	1.000000	1	1	1.0000000	NA
72 3	0.3257799	0.7184507	1	1	0.668878	1	1	0.7114198	1

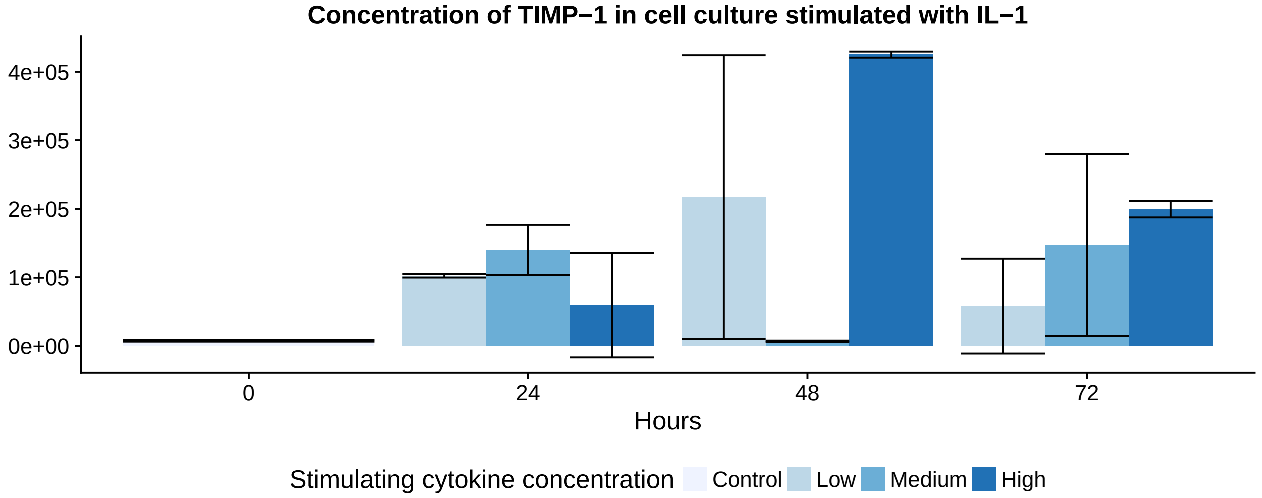

	Df	Sum Sq	Mean Sq	F value	Pr(>F)
Time	1	74425177544	74425177544	5.3586247	0.0313645
Concentration	1	52565337607	52565337607	3.7847127	0.0659153
Time:Concentration	1	4410030288	4410030288	0.3175229	0.5793639
Residuals	20	277777159720	13888857986	NA	NA

	0 0	24 1	24 2	24 3	48 1	48 2	48 3	72 1	72 2
24 1	1.0000000	NA	NA	NA	NA	NA	NA	NA	NA
24 2	1.0000000	1.0000000	NA	NA	NA	NA	NA	NA	NA
24 3	1.0000000	1.0000000	1.0000000	NA	NA	NA	NA	NA	NA
48 1	0.1402857	1.0000000	1.0000000	1.0000000	NA	NA	NA	NA	NA
48 2	1.0000000	1.0000000	1.0000000	1.0000000	0.5039109	NA	NA	NA	NA
48 3	0.0002400	0.0232867	0.0645901	0.0075932	0.5363679	0.0020436	NA	NA	NA
72 1	1.0000000	1.0000000	1.0000000	1.0000000	1.0000000	1.0000000	0.0073323	NA	NA
72 2	1.0000000	1.0000000	1.0000000	1.0000000	1.0000000	1.0000000	0.0788561	1	NA
72 3	0.2550642	1.0000000	1.0000000	1.0000000	1.0000000	0.8167651	0.3297217	1	1

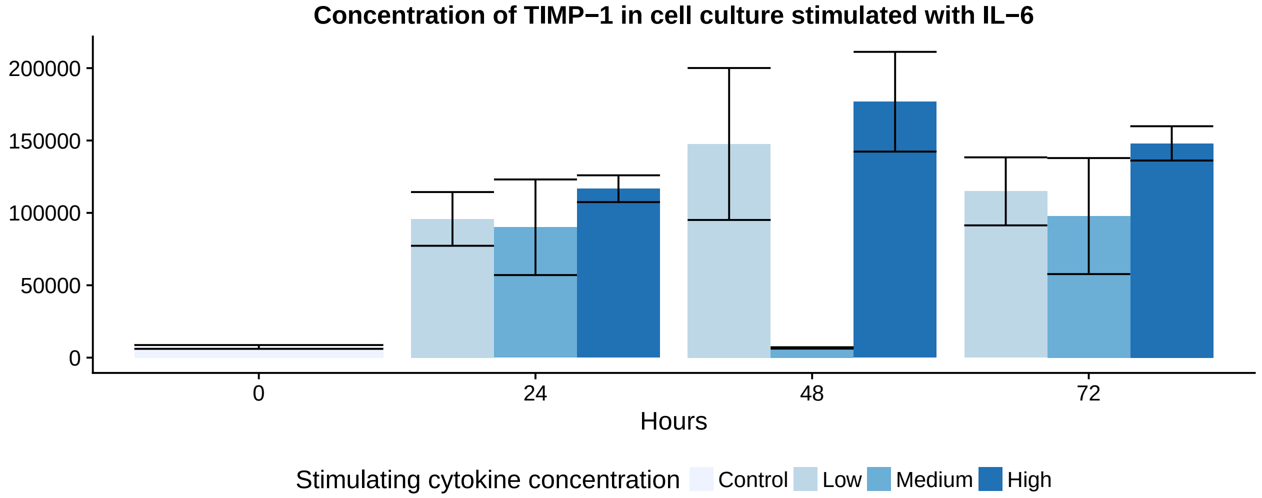

	Df	Sum Sq	Mean Sq	F value	Pr(>F)
Time	1	36332531182	36332531182	16.7135570	0.0005724
Concentration	1	11400805190	11400805190	5.2445564	0.0330175
Time:Concentration	1	1990511462	1990511462	0.9156677	0.3500400
Residuals	20	43476719170	2173835958	NA	NA

	0 0	24 1	24 2	24 3	48 1	48 2	48 3	72 1	72 2
24 1	0.0186430	NA	NA	NA	NA	NA	NA	NA	NA
24 2	0.0331499	1.0000000	NA	NA	NA	NA	NA	NA	NA
24 3	0.0025433	1.0000000	1.0000000	NA	NA	NA	NA	NA	NA
48 1	0.0001790	1.0000000	1.0000000	1.0000000	NA	NA	NA	NA	NA
48 2	1.0000000	0.0908446	0.1475698	0.0162848	0.001516	NA	NA	NA	NA
48 3	0.0000198	0.1804717	0.1109943	1.0000000	1.000000	0.0001994	NA	NA	NA
72 1	0.0030104	1.0000000	1.0000000	1.0000000	1.000000	0.0188783	0.8936572	NA	NA
72 2	0.0153578	1.0000000	1.0000000	1.0000000	1.000000	0.0770572	0.2130496	1	NA
72 3	0.0001732	1.0000000	1.0000000	1.0000000	1.000000	0.0014710	1.0000000	1	1

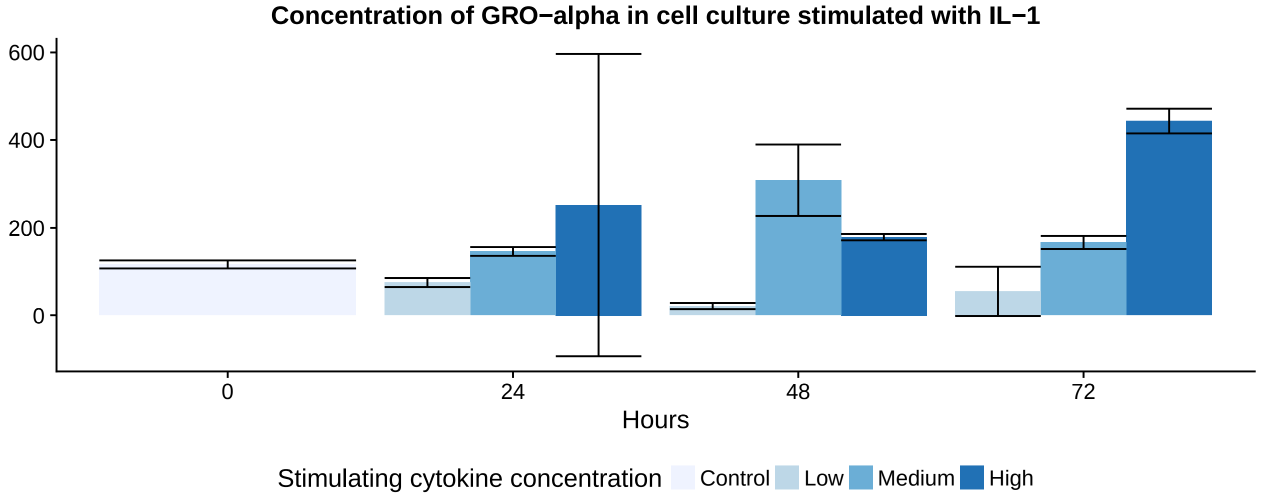

	Df	Sum Sq	Mean Sq	F value	Pr(>F)
Time	1	32354.62	32354.62	3.074146	0.0948610
Concentration	1	109923.09	109923.09	10.444244	0.0041816
Time:Concentration	1	86121.16	86121.16	8.182725	0.0096677
Residuals	20	210495.06	10524.75	NA	NA

	0 0	24 1	24 2	24 3	48 1	48 2	48 3	72 1	72 2
24 1	1.0000000	NA	NA	NA	NA	NA	NA	NA	NA
24 2	1.0000000	1.0000000	NA	NA	NA	NA	NA	NA	NA
24 3	1.0000000	1.0000000	1.0000000	NA	NA	NA	NA	NA	NA
48 1	1.0000000	1.0000000	1.0000000	1	NA	NA	NA	NA	NA
48 2	1.0000000	1.0000000	1.0000000	1	0.4535139	NA	NA	NA	NA
48 3	1.0000000	1.0000000	1.0000000	1	1.0000000	1.0000000	NA	NA	NA
72 1	1.0000000	1.0000000	1.0000000	1	1.0000000	0.9022218	1.000000	NA	NA
72 2	1.0000000	1.0000000	1.0000000	1	1.0000000	1.0000000	1.000000	1.0000000	NA
72 3	0.0440615	0.0851148	0.3647854	1	0.0287156	1.0000000	0.710474	0.0566696	0.5568131

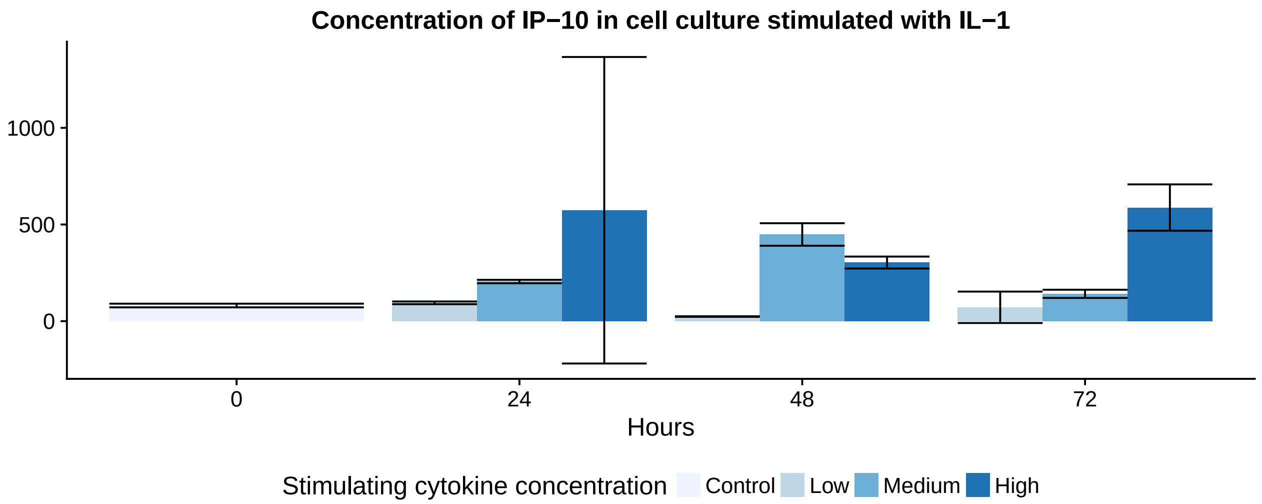

	Df	Sum Sq	Mean Sq	F value	Pr(>F)
Time	1	82861.10	82861.10	1.8903540	0.1843707
Concentration	1	575767.05	575767.05	13.1352769	0.0016903
Time:Concentration	1	39713.08	39713.08	0.9059953	0.3525417
Residuals	20	876672.87	43833.64	NA	NA

	0 0	24 1	24 2	24 3	48 1	48 2	48 3	72 1	72 2
24 1	1.0000000	NA	NA	NA	NA	NA	NA	NA	NA
24 2	1.0000000	1	NA	NA	NA	NA	NA	NA	NA
24 3	0.6500541	1	1	NA	NA	NA	NA	NA	NA
48 1	1.0000000	1	1	1	NA	NA	NA	NA	NA
48 2	1.0000000	1	1	1	1.000000	NA	NA	NA	NA
48 3	1.0000000	1	1	1	1.000000	1	NA	NA	NA
72 1	1.0000000	1	1	1	1.000000	1	1	NA	NA
72 2	1.0000000	1	1	1	1.000000	1	1	1	NA
72 3	0.5563380	1	1	1	0.931156	1	1	1	1

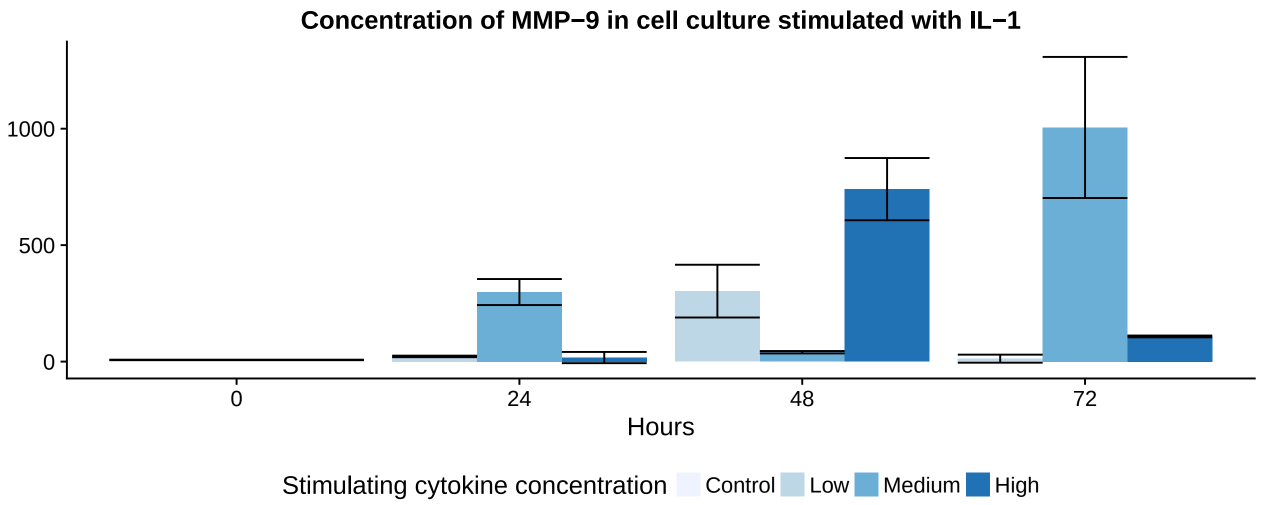

	Df	Sum Sq	Mean Sq	F value	Pr(>F)
Time	1	548809.06	548809.06	5.8119953	0.0256579
Concentration	1	63546.44	63546.44	0.6729692	0.4216885
Time:Concentration	1	33547.48	33547.48	0.3552744	0.5578288
Residuals	20	1888539.27	94426.96	NA	NA

	0 0	24 1	24 2	24 3	48 1	48 2	48 3	72 1	72 2
24 1	1.0000000	NA	NA	NA	NA	NA	NA	NA	NA
24 2	0.0959624	0.5150631	NA	NA	NA	NA	NA	NA	NA
24 3	1.0000000	1.0000000	0.4656068	NA	NA	NA	NA	NA	NA
48 1	0.0867999	0.4744968	1.0000000	0.4288822	NA	NA	NA	NA	NA
48 2	1.0000000	1.0000000	0.7490879	1.0000000	0.6905372	NA	NA	NA	NA
48 3	0.0000084	0.0001187	0.0169114	0.0001099	0.0182976	0.0001586	NA	NA	NA
72 1	1.0000000	1.0000000	0.4214815	1.0000000	0.3881970	1.0000000	0.0001020	NA	NA
72 2	0.0000002	0.0000028	0.0001429	0.0000026	0.0001522	0.0000035	0.6549916	2.4e-06	NA
72 3	1.0000000	1.0000000	1.0000000	1.0000000	1.0000000	1.0000000	0.0004898	1.0e+00	8.5e-06

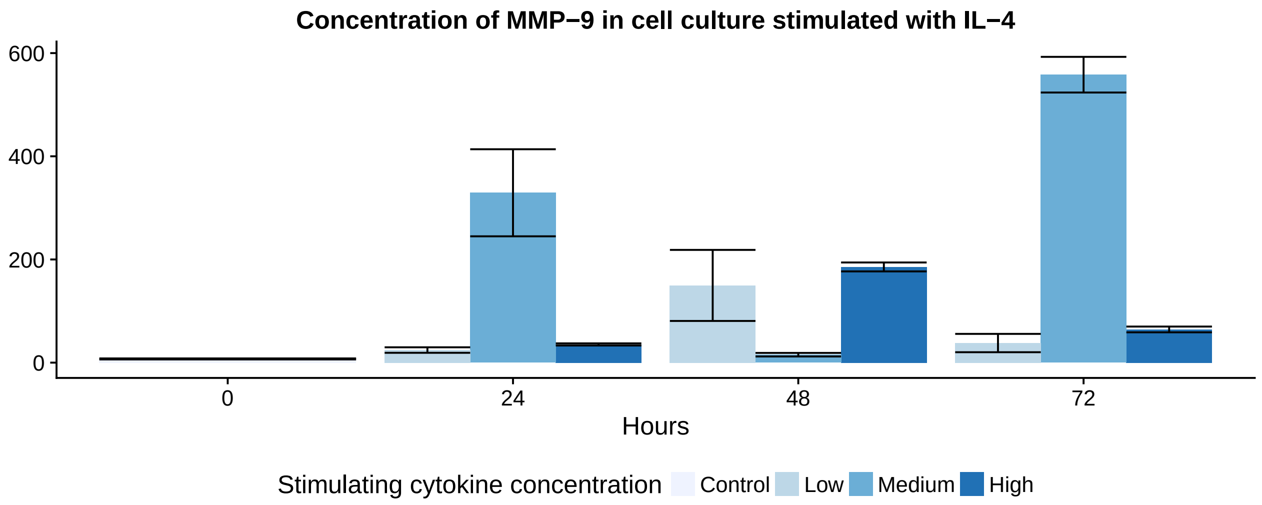

	Df	Sum Sq	Mean Sq	F value	Pr(>F)
Time	1	117604.4327	117604.4327	4.5134208	0.0462889
Concentration	1	6546.8396	6546.8396	0.2512545	0.6216656
Time:Concentration	1	550.2175	550.2175	0.0211162	0.8859173
Residuals	20	521132.1458	26056.6073	NA	NA

	0 0	24 1	24 2	24 3	48 1	48 2	48 3	72 1	72 2
24 1	1.0000000	NA	NA	NA	NA	NA	NA	NA	NA
24 2	0.0000002	0.0000053	NA	NA	NA	NA	NA	NA	NA
24 3	1.0000000	1.0000000	0.0000084	NA	NA	NA	NA	NA	NA
48 1	0.0028797	0.0558491	0.0021548	0.1115935	NA	NA	NA	NA	NA
48 2	1.0000000	1.0000000	0.0000037	1.0000000	0.0318674	NA	NA	NA	NA
48 3	0.0002698	0.0062376	0.0177509	0.0119596	1.0000000	0.0037002	NA	NA	NA
72 1	1.0000000	1.0000000	0.0000094	1.0000000	0.1323263	1.0000000	0.0140576	NA	NA
72 2	0.0000000	0.0000000	0.0001593	0.0000000	0.0000001	0.0000000	0.0000004	0	NA
72 3	1.0000000	1.0000000	0.0000293	1.0000000	0.7121068	1.0000000	0.0723419	1	0

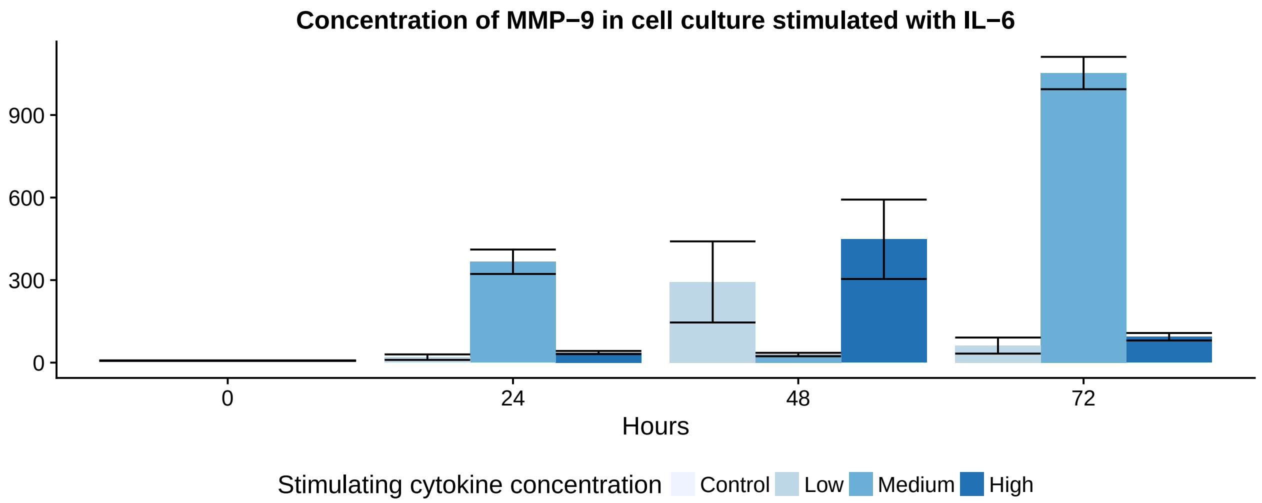

	Df	Sum Sq	Mean Sq	F value	Pr(>F)
Time	1	509457.370	509457.370	6.2253492	0.0214584
Concentration	1	8382.614	8382.614	0.1024319	0.7522500
Time:Concentration	1	4597.537	4597.537	0.0561799	0.8150498
Residuals	20	1636719.006	81835.950	NA	NA

	0 0	24 1	24 2	24 3	48 1	48 2	48 3	72 1	72 2
24 1	1.0000000	NA	NA	NA	NA	NA	NA	NA	NA
24 2	0.0001442	0.0018957	NA	NA	NA	NA	NA	NA	NA
24 3	1.0000000	1.0000000	0.0031237	NA	NA	NA	NA	NA	NA
48 1	0.0017001	0.0181046	1.0000000	0.0311955	NA	NA	NA	NA	NA
48 2	1.0000000	1.0000000	0.0025085	1.0000000	0.0245751	NA	NA	NA	NA
48 3	0.0000130	0.0001964	1.0000000	0.0003068	0.9154610	0.0002522	NA	NA	NA
72 1	1.0000000	1.0000000	0.0067384	1.0000000	0.0715169	1.0000000	0.0006106	NA	NA
72 2	0.0000000	0.0000000	0.0000007	0.0000000	0.0000002	0.0000000	0.0000033	0	NA
72 3	1.0000000	1.0000000	0.0186460	1.0000000	0.2102318	1.0000000	0.0015275	1	0

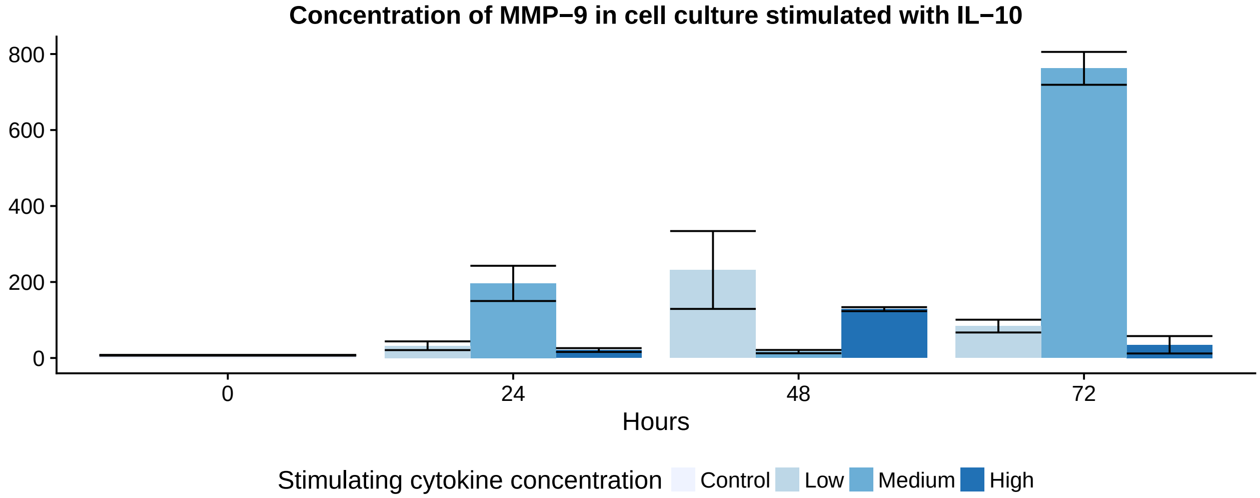

	Df	Sum Sq	Mean Sq	F value	Pr(>F)
Time	1	244066.7682	244066.7682	6.3550905	0.0203042
Concentration	1	13435.0660	13435.0660	0.3498267	0.5608377
Time:Concentration	1	348.0532	348.0532	0.0090627	0.9251047
Residuals	20	768098.4817	38404.9241	NA	NA

	0 0	24 1	24 2	24 3	48 1	48 2	48 3	72 1	72 2
24 1	1.0000000	NA	NA	NA	NA	NA	NA	NA	NA
24 2	0.0003024	0.0100447	NA	NA	NA	NA	NA	NA	NA
24 3	1.0000000	1.0000000	0.0053740	NA	NA	NA	NA	NA	NA
48 1	0.0000429	0.0015014	1.0000000	0.0008436	NA	NA	NA	NA	NA
48 2	1.0000000	1.0000000	0.0042852	1.0000000	0.0006851	NA	NA	NA	NA
48 3	0.0241806	0.5334113	1.0000000	0.2719491	0.3561889	0.212077	NA	NA	NA
72 1	0.6109744	1.0000000	0.2044959	1.0000000	0.0255269	1.000000	1.0000000	NA	NA
72 2	0.0000000	0.0000000	0.0000000	0.0000000	0.0000000	0.000000	0.0000000	0	NA
72 3	1.0000000	1.0000000	0.0115847	1.0000000	0.0017130	1.000000	0.6200953	1	0

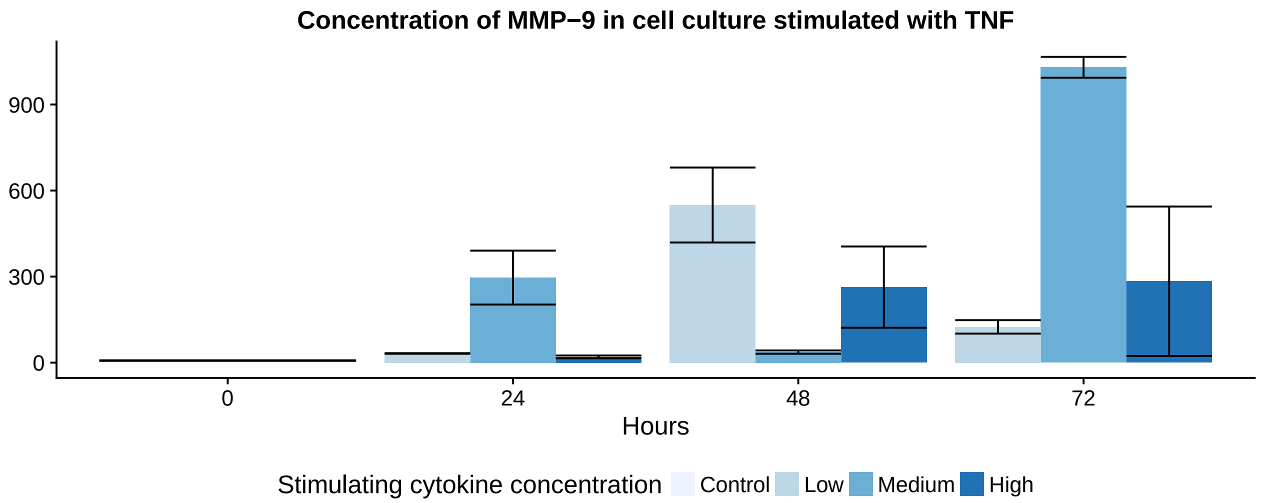

	Df	Sum Sq	Mean Sq	F value	Pr(>F)
Time	1	752279.046	752279.046	10.7571503	0.0037459
Concentration	1	16186.014	16186.014	0.2314505	0.6356717
Time:Concentration	1	8924.826	8924.826	0.1276198	0.7246547
Residuals	20	1398658.617	69932.931	NA	NA

	0 0	24 1	24 2	24 3	48 1	48 2	48 3	72 1	72 2
24 1	1.0000000	NA	NA	NA	NA	NA	NA	NA	NA
24 2	0.0727926	0.5153334	NA	NA	NA	NA	NA	NA	NA
24 3	1.0000000	1.0000000	0.3976580	NA	NA	NA	NA	NA	NA
48 1	0.0001755	0.0025128	0.6616581	0.0019976	NA	NA	NA	NA	NA
48 2	1.0000000	1.0000000	0.5712846	1.0000000	0.0027554	NA	NA	NA	NA
48 3	0.1767082	1.0000000	1.0000000	0.8177962	0.3208734	1.0000000	NA	NA	NA
72 1	1.0000000	1.0000000	1.0000000	1.0000000	0.0162258	1.0000000	1.00e+00	NA	NA
72 2	0.0000001	0.0000013	0.0000565	0.0000011	0.0052498	0.0000014	3.35e-05	4.5e-06	NA
72 3	0.1024902	0.6811624	1.0000000	0.5262902	0.5004937	0.7546351	1.00e+00	1.0e+00	4.61e-05

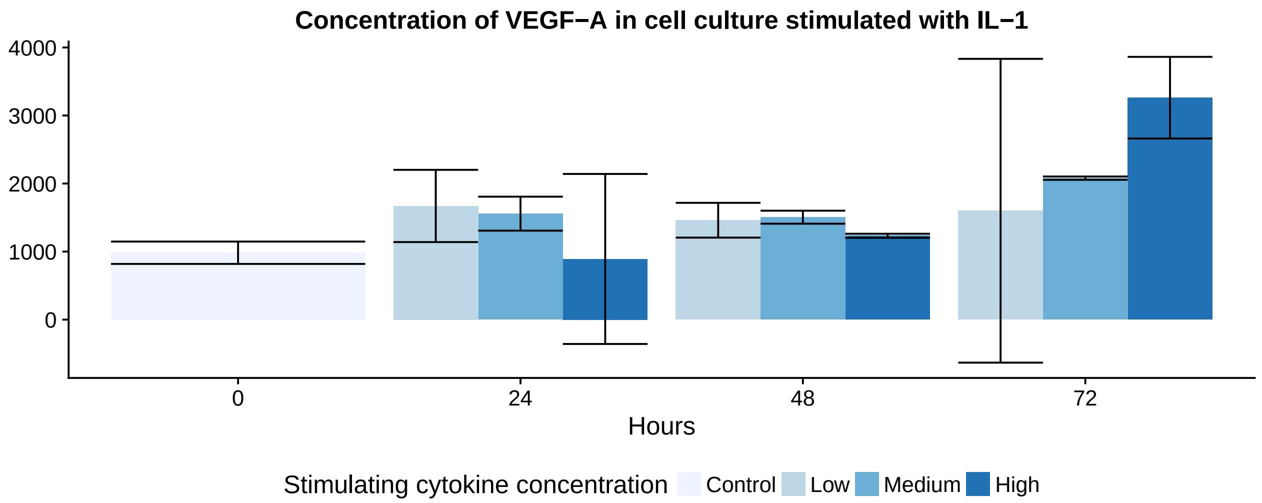

	Df	Sum Sq	Mean Sq	F value	Pr(>F)
Time	1	4846541.88	4846541.88	9.4771343	0.0059268
Concentration	1	12376.65	12376.65	0.0242018	0.8779319
Time:Concentration	1	1825698.16	1825698.16	3.5700479	0.0734162
Residuals	20	10227863.68	511393.18	NA	NA

	0 0	24 1	24 2	24 3	48 1	48 2	48 3	72 1	72 2
24 1	1.0000000	NA	NA	NA	NA	NA	NA	NA	NA
24 2	1.0000000	1	NA	NA	NA	NA	NA	NA	NA
24 3	1.0000000	1	1	NA	NA	NA	NA	NA	NA
48 1	1.0000000	1	1	1.0000000	NA	NA	NA	NA	NA
48 2	1.0000000	1	1	1.0000000	1	NA	NA	NA	NA
48 3	1.0000000	1	1	1.0000000	1	1	NA	NA	NA
72 1	1.0000000	1	1	1.0000000	1	1	1.0000000	NA	NA
72 2	1.0000000	1	1	1.0000000	1	1	1.0000000	1	NA
72 3	0.0836705	1	1	0.2628804	1	1	0.6618523	1	1

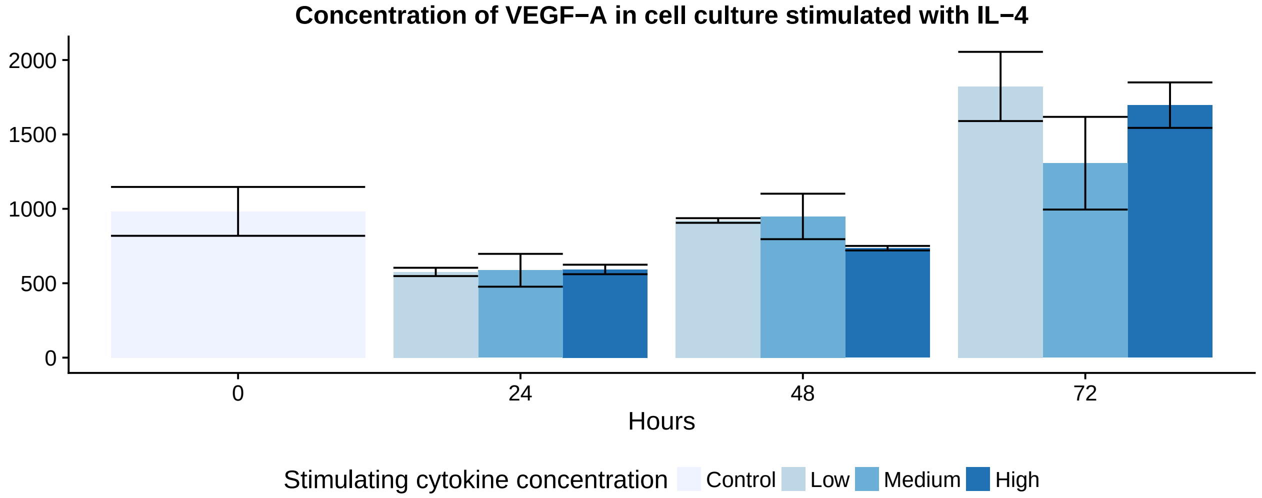

	Df	Sum Sq	Mean Sq	F value	Pr(>F)
Time	1	1400117.0	1400117.0	22.831504	0.0001146
Concentration	1	891577.1	891577.1	14.538818	0.0010890
Time:Concentration	1	528698.2	528698.2	8.621405	0.0081652
Residuals	20	1226478.1	61323.9	NA	NA

	0 0	24 1	24 2	24 3	48 1	48 2	48 3	72 1	72 2
24 1	0.3093438	NA	NA	NA	NA	NA	NA	NA	NA
24 2	0.3673816	1.0000000	NA	NA	NA	NA	NA	NA	NA
24 3	0.3996575	1.0000000	1.0000000	NA	NA	NA	NA	NA	NA
48 1	1.0000000	1.0000000	1.0000000	1.0000000	NA	NA	NA	NA	NA
48 2	1.0000000	1.0000000	1.0000000	1.0000000	1.0000000	NA	NA	NA	NA
48 3	1.0000000	1.0000000	1.0000000	1.0000000	1.0000000	1.0000000	NA	NA	NA
72 1	0.0006002	0.0000696	0.0000772	0.0000813	0.0023759	0.0032321	0.0003282	NA	NA
72 2	1.0000000	0.0171947	0.0196685	0.0210126	1.0000000	1.0000000	0.1238354	0.2481932	NA
72 3	0.0032031	0.0002332	0.0002606	0.0002753	0.0100851	0.0139724	0.0012245	1.0000000	1

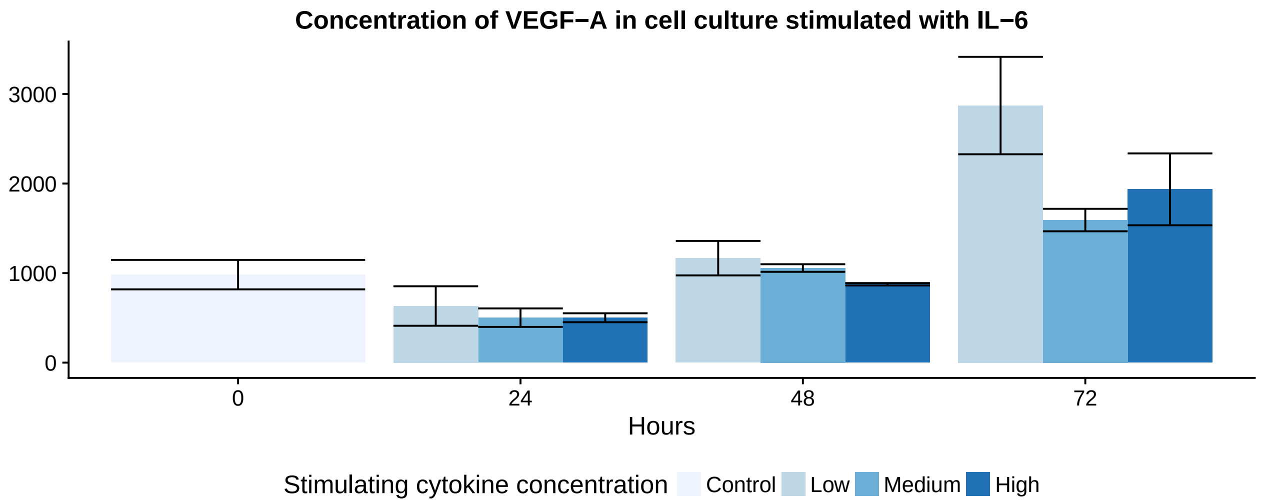

	Df	Sum Sq	Mean Sq	F value	Pr(>F)
Time	1	4653248.0	4653248.0	31.122142	0.0000185
Concentration	1	2942213.8	2942213.8	19.678297	0.0002540
Time:Concentration	1	213069.2	213069.2	1.425063	0.2465398
Residuals	20	2990313.4	149515.7	NA	NA

	0 0	24 1	24 2	24 3	48 1	48 2	48 3	72 1	72 2
24 1	1.0000000	NA	NA	NA	NA	NA	NA	NA	NA
24 2	0.9149730	1.0000000	NA	NA	NA	NA	NA	NA	NA
24 3	0.9073312	1.0000000	1.0000000	NA	NA	NA	NA	NA	NA
48 1	1.0000000	1.0000000	0.4708178	0.4675536	NA	NA	NA	NA	NA
48 2	1.0000000	1.0000000	1.0000000	1.0000000	1.0000000	NA	NA	NA	NA
48 3	1.0000000	1.0000000	1.0000000	1.0000000	1.0000000	1.0000000	NA	NA	NA
72 1	0.0000030	0.0000045	0.0000022	0.0000022	0.0001152	0.0000559	0.0000181	NA	NA
72 2	0.2278057	0.0351160	0.0116570	0.0115806	1.0000000	1.0000000	0.2956480	0.0025346	NA
72 3	0.0061890	0.0020939	0.0007799	0.0007754	0.1872219	0.0708993	0.0149465	0.0433820	1

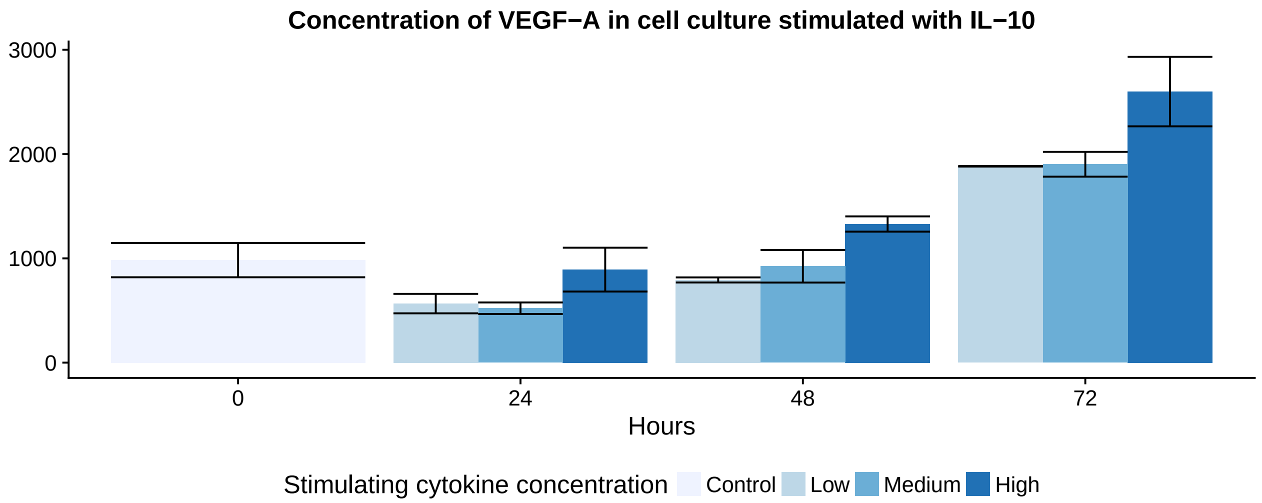

	Df	Sum Sq	Mean Sq	F value	Pr(>F)
Time	1	4310590.72	4310590.72	49.0832337	0.0000008
Concentration	1	63338.17	63338.17	0.7212102	0.4057982
Time:Concentration	1	2761571.34	2761571.34	31.4450756	0.0000173
Residuals	20	1756441.21	87822.06	NA	NA

	0 0	24 1	24 2	24 3	48 1	48 2	48 3	72 1	72 2
24 1	0.2636024	NA	NA	NA	NA	NA	NA	NA	NA
24 2	0.1326048	1.0000000	NA	NA	NA	NA	NA	NA	NA
24 3	1.0000000	1.0000000	1.0000000	NA	NA	NA	NA	NA	NA
48 1	1.0000000	1.0000000	1.0000000	1.0000000	NA	NA	NA	NA	NA
48 2	1.0000000	1.0000000	1.0000000	1.0000000	1.0000000	NA	NA	NA	NA
48 3	0.7809441	0.0115199	0.0068254	0.6616443	0.1903207	0.9885304	NA	NA	NA
72 1	0.0002778	0.0000362	0.0000244	0.0008782	0.0003145	0.0012429	0.1530220	NA	NA
72 2	0.0002191	0.0000304	0.0000206	0.0007148	0.0002587	0.0010082	0.1197965	1.0000000	NA
72 3	0.0000002	0.0000002	0.0000001	0.0000015	0.0000007	0.0000019	0.0000552	0.0201628	0.0255325

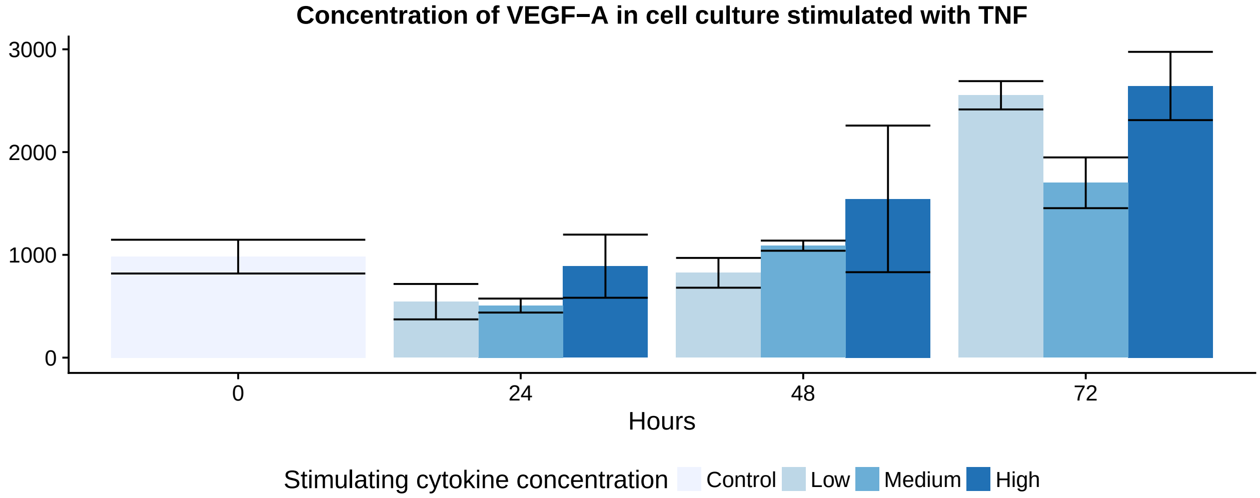

	Df	Sum Sq	Mean Sq	F value	Pr(>F)
Time	1	5952331.0	5952331.0	30.013746	0.0000231
Concentration	1	265115.6	265115.6	1.336806	0.2612230
Time:Concentration	1	1847841.1	1847841.1	9.317465	0.0062864
Residuals	20	3966403.3	198320.2	NA	NA

	0 0	24 1	24 2	24 3	48 1	48 2	48 3	72 1	72 2
24 1	1.0000000	NA	NA	NA	NA	NA	NA	NA	NA
24 2	1.0000000	1.0000000	NA	NA	NA	NA	NA	NA	NA
24 3	1.0000000	1.0000000	1.0000000	NA	NA	NA	NA	NA	NA
48 1	1.0000000	1.0000000	1.0000000	1.0000000	NA	NA	NA	NA	NA
48 2	1.0000000	1.0000000	1.0000000	1.0000000	1.0000000	NA	NA	NA	NA
48 3	0.9570192	0.0932554	0.0704117	1.0000000	0.7598724	1.0000000	NA	NA	NA
72 1	0.0001916	0.0001169	0.0000944	0.0009312	0.0006211	0.0034349	0.0880711	NA	NA
72 2	0.2296640	0.0294383	0.0223882	0.3832890	0.2363566	1.0000000	1.0000000	0.2840839	NA
72 3	0.0001014	0.0000704	0.0000572	0.0005289	0.0003568	0.0018829	0.0450960	1.0000000	0.144186

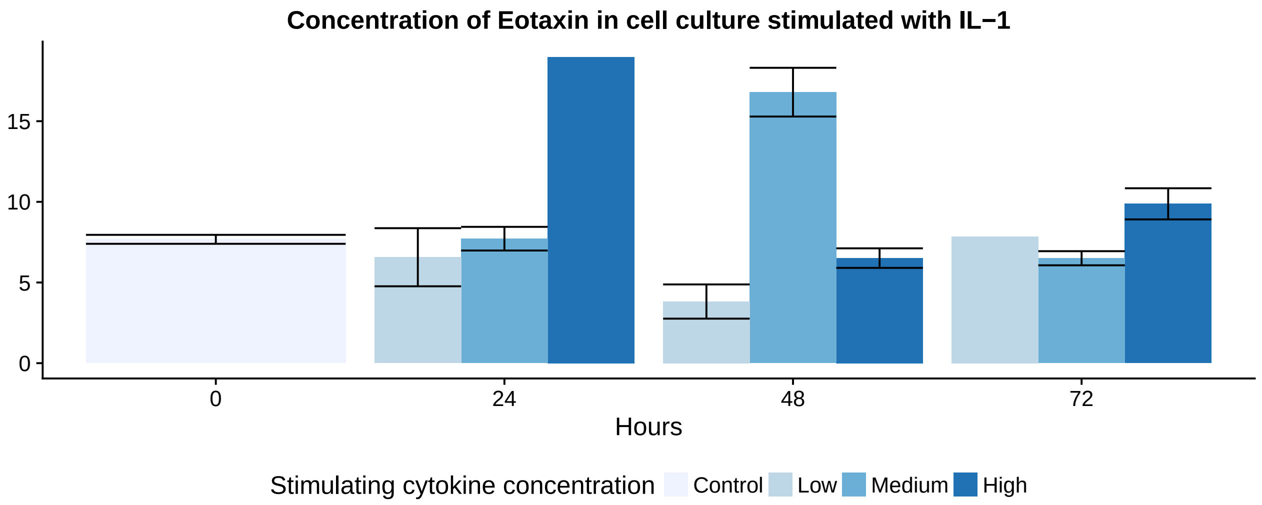

	Df	Sum Sq	Mean Sq	F value	Pr(>F)
Time	1	0.5122848	0.5122848	0.0354134	0.8528373
Concentration	1	71.1007953	71.1007953	4.9150813	0.0397376
Time:Concentration	1	1.2774965	1.2774965	0.0883112	0.7697373
Residuals	18	260.3851798	14.4658433	NA	NA

	0 0	24 1	24 2	24 3	48 1	48 2	48 3	72 1	72 2
24 1	NA	NA	NA	NA	NA	NA	NA	NA	NA
24 2	NA	NA	NA	NA	NA	NA	NA	NA	NA
24 3	NA	NA	NA	NA	NA	NA	NA	NA	NA
48 1	NA	NA	NA	NA	NA	NA	NA	NA	NA
48 2	NA	NA	NA	NA	NA	NA	NA	NA	NA
48 3	NA	NA	NA	NA	NA	NA	NA	NA	NA
72 1	NA	NA	NA	NA	NA	NA	NA	NA	NA
72 2	NA	NA	NA	NA	NA	NA	NA	NA	NA
72 3	NA	NA	NA	NA	NA	NA	NA	NA	NA

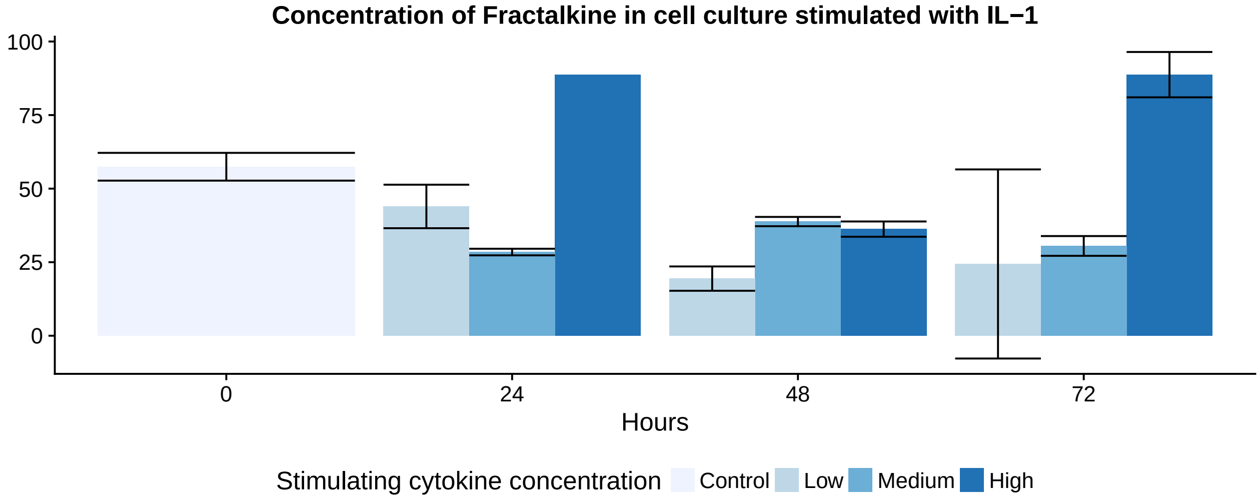

	Df	Sum Sq	Mean Sq	F value	Pr(>F)
Time	1	578.6542	578.6542	1.902765	0.1837900
Concentration	1	1476.4940	1476.4940	4.855095	0.0401029
Time:Concentration	1	3468.7910	3468.7910	11.406285	0.0031626
Residuals	19	5778.1329	304.1123	NA	NA

	0 0	24 1	24 2	24 3	48 1	48 2	48 3	72 1	72 2
24 1	NA	NA	NA	NA	NA	NA	NA	NA	NA
24 2	NA	NA	NA	NA	NA	NA	NA	NA	NA
24 3	NA	NA	NA	NA	NA	NA	NA	NA	NA
48 1	NA	NA	NA	NA	NA	NA	NA	NA	NA
48 2	NA	NA	NA	NA	NA	NA	NA	NA	NA
48 3	NA	NA	NA	NA	NA	NA	NA	NA	NA
72 1	NA	NA	NA	NA	NA	NA	NA	NA	NA
72 2	NA	NA	NA	NA	NA	NA	NA	NA	NA
72 3	NA	NA	NA	NA	NA	NA	NA	NA	NA

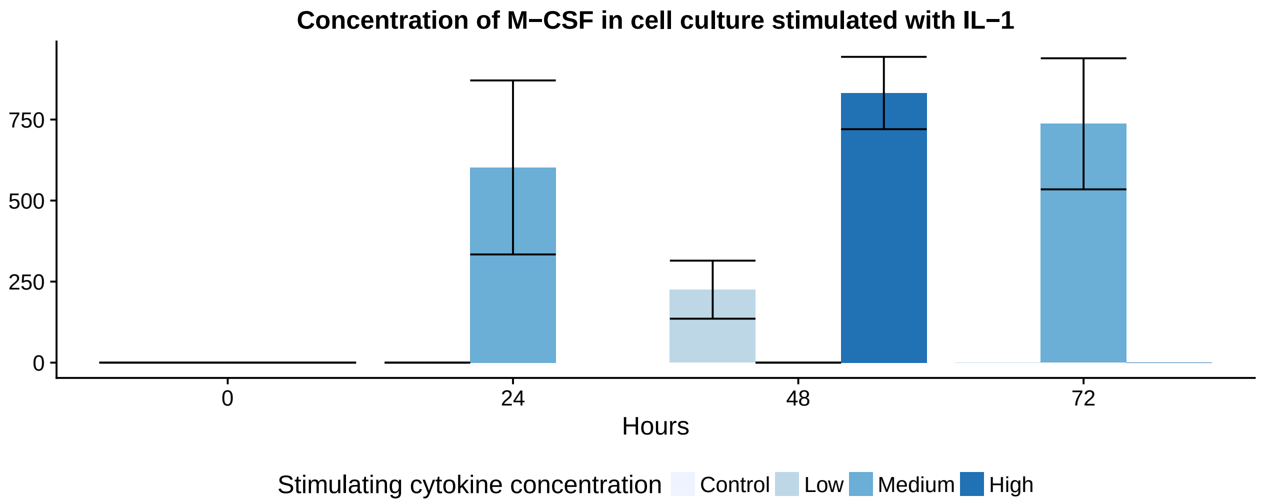

	Df	Sum Sq	Mean Sq	F value	Pr(>F)
Time	1	281500.38	281500.38	3.0397668	0.1031556
Concentration	1	552185.19	552185.19	5.9627422	0.0284856
Time:Concentration	1	24006.82	24006.82	0.2592364	0.6185753
Residuals	14	1296482.80	92605.91	NA	NA

	0 0	24 1	24 2	24 3	48 1	48 2	48 3	72 1	72 2
24 1	NA	NA	NA	NA	NA	NA	NA	NA	NA
24 2	NA	NA	NA	NA	NA	NA	NA	NA	NA
24 3	NaN	NaN	NaN	NA	NA	NA	NA	NA	NA
48 1	NA	NA	NA	NaN	NA	NA	NA	NA	NA
48 2	NA	NA	NA	NaN	NA	NA	NA	NA	NA
48 3	NA	NA	NA	NaN	NA	NA	NA	NA	NA
72 1	NA	NA	NA	NaN	NA	NA	NA	NA	NA
72 2	NA	NA	NA	NaN	NA	NA	NA	NA	NA
72 3	NA	NA	NA	NaN	NA	NA	NA	NA	NA

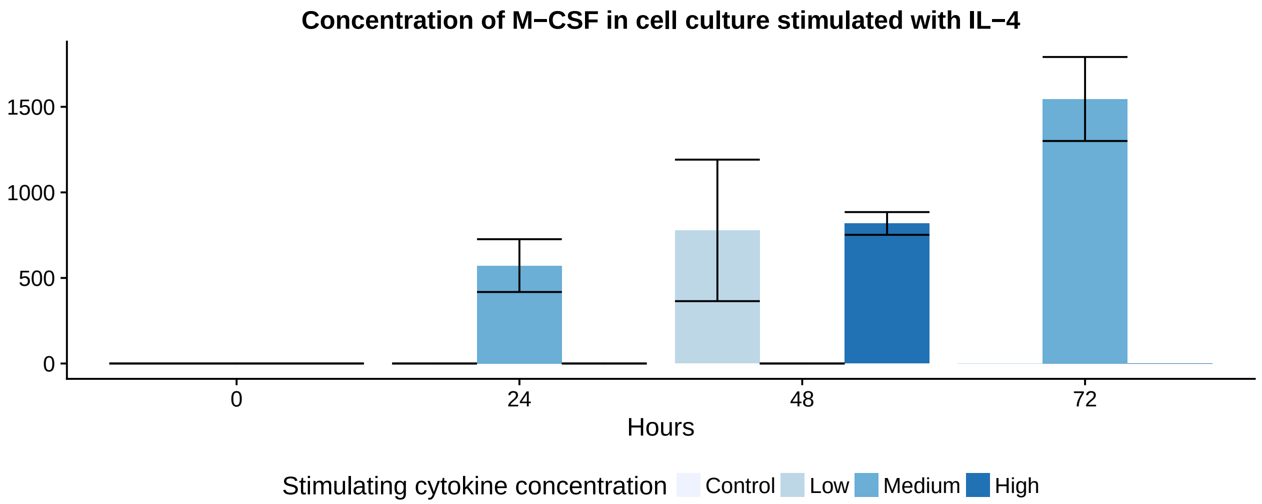

	Df	Sum Sq	Mean Sq	F value	Pr(>F)
Time	1	1526461.991	1526461.991	6.1864690	0.0242884
Concentration	1	2095.968	2095.968	0.0084946	0.9277103
Time:Concentration	1	7676.040	7676.040	0.0311096	0.8622093
Residuals	16	3947872.689	246742.043	NA	NA

	0 0	24 1	24 2	24 3	48 1	48 2	48 3	72 1	72 2
24 1	NA	NA	NA	NA	NA	NA	NA	NA	NA
24 2	NA	NA	NA	NA	NA	NA	NA	NA	NA
24 3	NA	NA	NA	NA	NA	NA	NA	NA	NA
48 1	NA	NA	NA	NA	NA	NA	NA	NA	NA
48 2	NA	NA	NA	NA	NA	NA	NA	NA	NA
48 3	NA	NA	NA	NA	NA	NA	NA	NA	NA
72 1	NA	NA	NA	NA	NA	NA	NA	NA	NA
72 2	NA	NA	NA	NA	NA	NA	NA	NA	NA
72 3	NA	NA	NA	NA	NA	NA	NA	NA	NA

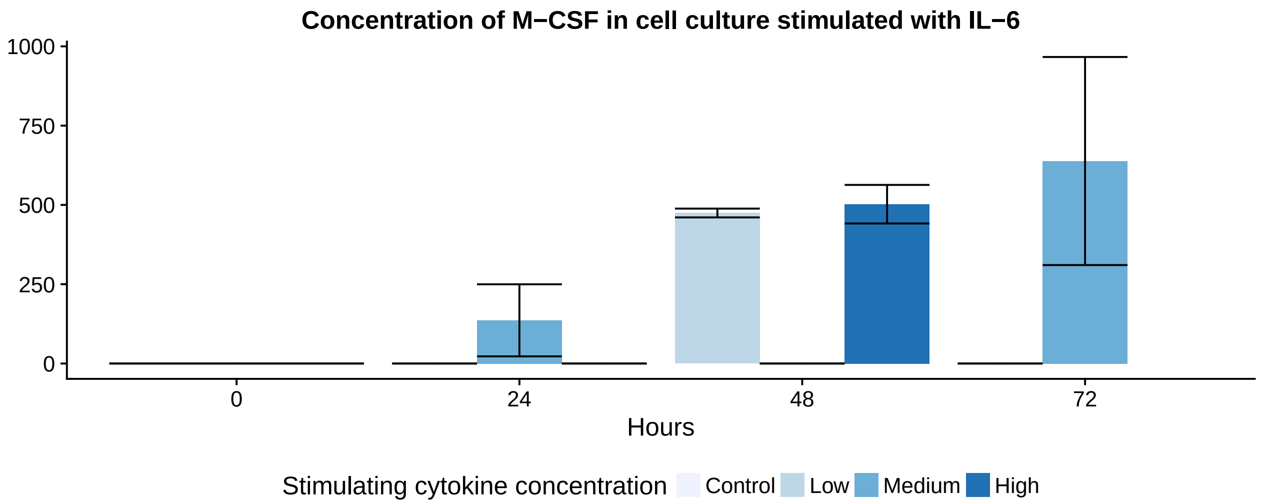

	Df	Sum Sq	Mean Sq	F value	Pr(>F)
Time	1	361637.60	361637.60	8.2949354	0.0108812
Concentration	1	22852.15	22852.15	0.5241632	0.4795241
Time:Concentration	1	235997.42	235997.42	5.4131080	0.0334461
Residuals	16	697558.37	43597.40	NA	NA

	0 0	24 1	24 2	24 3	48 1	48 2	48 3	72 1	72 2
24 1	NA	NA	NA	NA	NA	NA	NA	NA	NA
24 2	NA	NA	NA	NA	NA	NA	NA	NA	NA
24 3	NA	NA	NA	NA	NA	NA	NA	NA	NA
48 1	NA	NA	NA	NA	NA	NA	NA	NA	NA
48 2	NA	NA	NA	NA	NA	NA	NA	NA	NA
48 3	NA	NA	NA	NA	NA	NA	NA	NA	NA
72 1	NA	NA	NA	NA	NA	NA	NA	NA	NA
72 2	NA	NA	NA	NA	NA	NA	NA	NA	NA
72 3	NaN	NaN	NaN	NaN	NaN	NaN	NaN	NaN	NaN

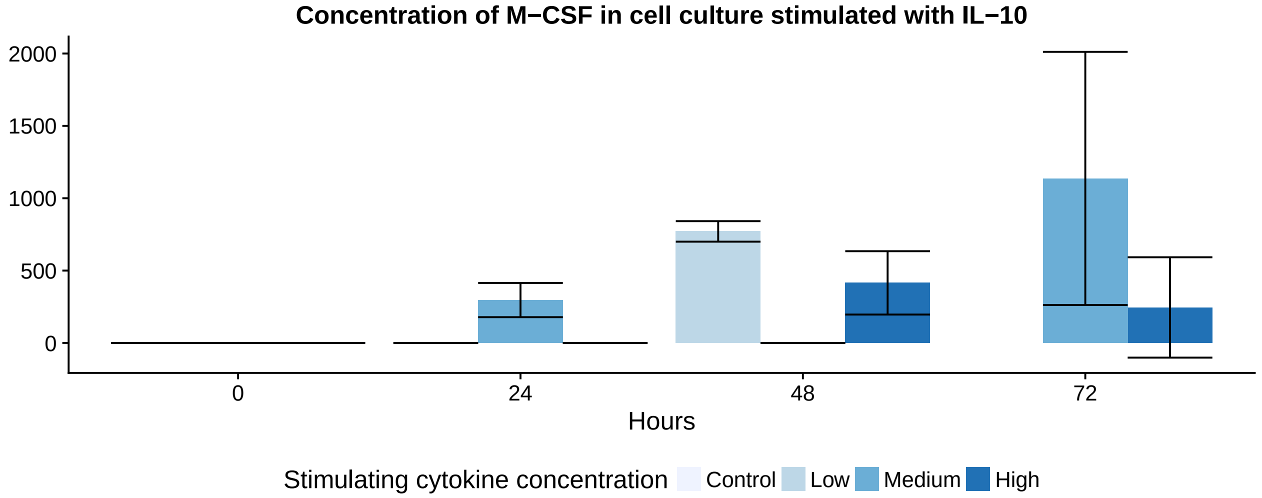

	Df	Sum Sq	Mean Sq	F value	Pr(>F)
Time	1	1207311.2	1207311.2	9.512101	0.0071118
Concentration	1	326713.7	326713.7	2.574095	0.1281806
Time:Concentration	1	159773.7	159773.7	1.258817	0.2784313
Residuals	16	2030779.4	126923.7	NA	NA

	0 0	24 1	24 2	24 3	48 1	48 2	48 3	72 1	72 2
24 1	NA	NA	NA	NA	NA	NA	NA	NA	NA
24 2	NA	NA	NA	NA	NA	NA	NA	NA	NA
24 3	NA	NA	NA	NA	NA	NA	NA	NA	NA
48 1	NA	NA	NA	NA	NA	NA	NA	NA	NA
48 2	NA	NA	NA	NA	NA	NA	NA	NA	NA
48 3	NA	NA	NA	NA	NA	NA	NA	NA	NA
72 1	NaN	NaN	NaN	NaN	NaN	NaN	NaN	NA	NA
72 2	NA	NA	NA	NA	NA	NA	NA	NaN	NA
72 3	NA	NA	NA	NA	NA	NA	NA	NaN	NA

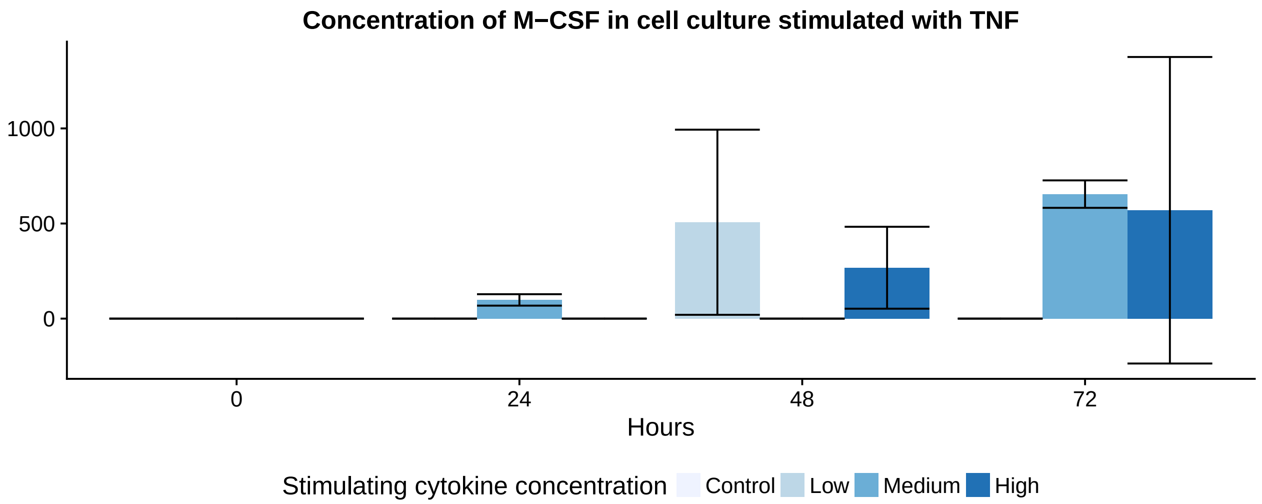

	Df	Sum Sq	Mean Sq	F value	Pr(>F)
Time	1	568583.449	568583.449	6.5767290	0.0194985
Concentration	1	2545.657	2545.657	0.0294453	0.8656693
Time:Concentration	1	194039.596	194039.596	2.2444302	0.1514286
Residuals	18	1556169.044	86453.836	NA	NA

	0 0	24 1	24 2	24 3	48 1	48 2	48 3	72 1	72 2
24 1	1.0000000	NA	NA	NA	NA	NA	NA	NA	NA
24 2	1.0000000	1	NA	NA	NA	NA	NA	NA	NA
24 3	1.0000000	1	1	NA	NA	NA	NA	NA	NA
48 1	1.0000000	1	1	1	NA	NA	NA	NA	NA
48 2	1.0000000	1	1	1	1	NA	NA	NA	NA
48 3	1.0000000	1	1	1	1	1	NA	NA	NA
72 1	1.0000000	1	1	1	1	1	1	NA	NA
72 2	0.8644204	1	1	1	1	1	1	1	NA
72 3	1.0000000	1	1	1	1	1	1	1	1

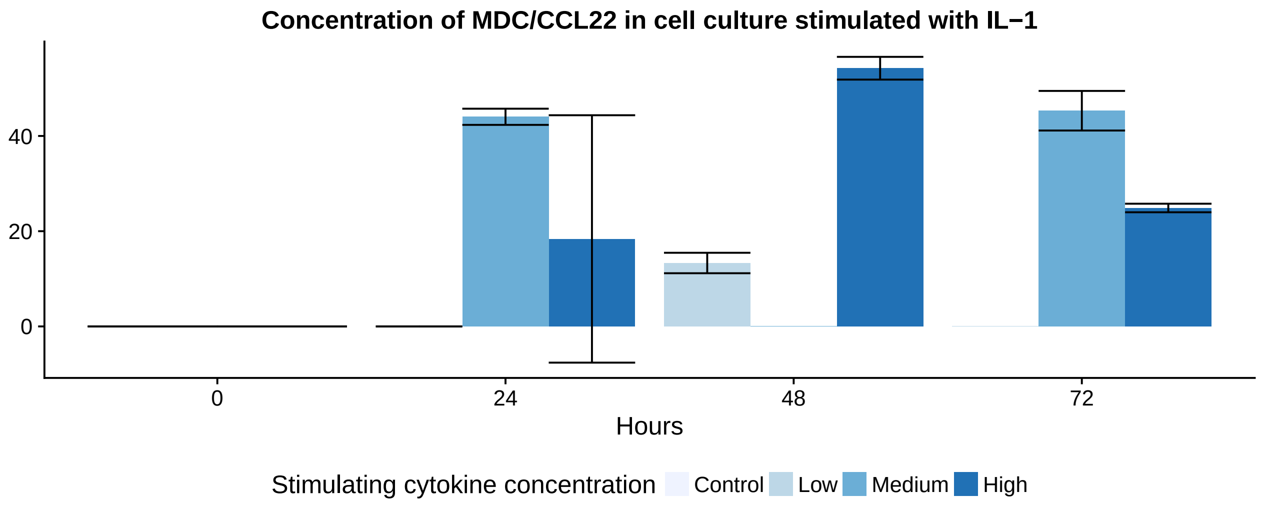

	Df	Sum Sq	Mean Sq	F value	Pr(>F)
Time	1	911.91310	911.91310	2.6085762	0.1285916
Concentration	1	2111.41310	2111.41310	6.0398101	0.0276365
Time:Concentration	1	18.92932	18.92932	0.0541483	0.8193635
Residuals	14	4894.15776	349.58270	NA	NA

	0 0	24 1	24 2	24 3	48 1	48 2	48 3	72 1	72 2
24 1	NA	NA	NA	NA	NA	NA	NA	NA	NA
24 2	NA	NA	NA	NA	NA	NA	NA	NA	NA
24 3	NA	NA	NA	NA	NA	NA	NA	NA	NA
48 1	NA	NA	NA	NA	NA	NA	NA	NA	NA
48 2	NA	NA	NA	NA	NA	NA	NA	NA	NA
48 3	NA	NA	NA	NA	NA	NA	NA	NA	NA
72 1	NA	NA	NA	NA	NA	NA	NA	NA	NA
72 2	NA	NA	NA	NA	NA	NA	NA	NA	NA
72 3	NA	NA	NA	NA	NA	NA	NA	NA	NA

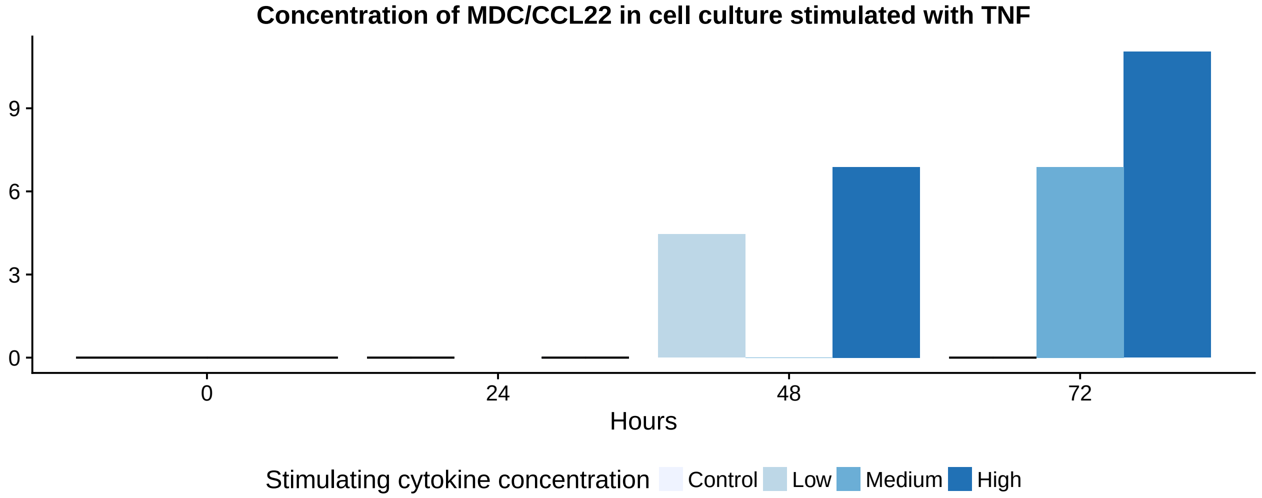

	Df	Sum Sq	Mean Sq	F value	Pr(>F)
Time	1	49.04796	49.047961	13.782839	0.0048261
Concentration	1	16.67748	16.677482	4.686495	0.0586047
Time:Concentration	1	72.72053	72.720529	20.435005	0.0014457
Residuals	9	32.02763	3.558626	NA	NA

	0 0	24 1	24 2	24 3	48 1	48 2	48 3	72 1	72 2
24 1	NA	NA	NA	NA	NA	NA	NA	NA	NA
24 2	NaN	NaN	NA	NA	NA	NA	NA	NA	NA
24 3	NA	NA	NaN	NA	NA	NA	NA	NA	NA
48 1	NA	NA	NaN	NA	NA	NA	NA	NA	NA
48 2	NA	NA	NaN	NA	NA	NA	NA	NA	NA
48 3	NA	NA	NaN	NA	NA	NA	NA	NA	NA
72 1	NA	NA	NaN	NA	NA	NA	NA	NA	NA
72 2	NA	NA	NaN	NA	NA	NA	NA	NA	NA
72 3	NA	NA	NaN	NA	NA	NA	NA	NA	NA

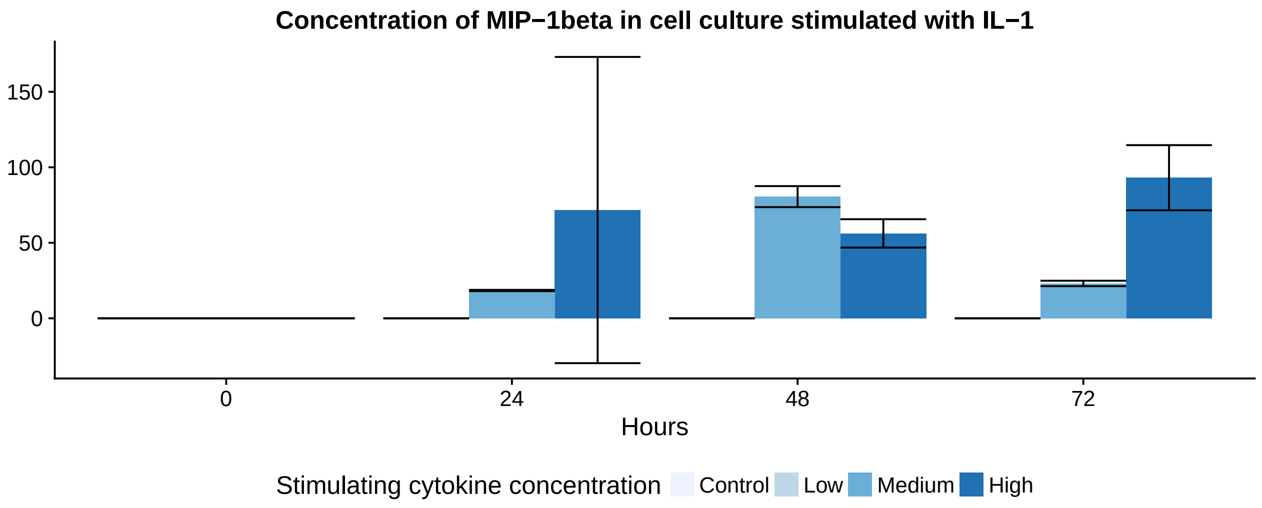

	Df	Sum Sq	Mean Sq	F value	Pr(>F)
Time	1	5206.589	5206.5888	6.185414	0.0218284
Concentration	1	15642.512	15642.5116	18.583263	0.0003401
Time:Concentration	1	2249.935	2249.9352	2.672917	0.1177111
Residuals	20	16835.054	841.7527	NA	NA

	0 0	24 1	24 2	24 3	48 1	48 2	48 3	72 1	72 2
24 1	1.0000000	NA	NA	NA	NA	NA	NA	NA	NA
24 2	1.0000000	1.0000000	NA	NA	NA	NA	NA	NA	NA
24 3	0.3197657	0.9984661	1.0000000	NA	NA	NA	NA	NA	NA
48 1	1.0000000	1.0000000	1.0000000	0.9984661	NA	NA	NA	NA	NA
48 2	0.1471614	0.5346058	1.0000000	1.0000000	0.5346058	NA	NA	NA	NA
48 3	1.0000000	1.0000000	1.0000000	1.0000000	1.0000000	1.0000000	NA	NA	NA
72 1	1.0000000	1.0000000	1.0000000	0.9984661	1.0000000	0.5346058	1	NA	NA
72 2	1.0000000	1.0000000	1.0000000	1.0000000	1.0000000	1.0000000	1	1.0000000	NA
72 3	0.0497855	0.2193896	0.8108449	1.0000000	0.2193896	1.0000000	1	0.2193896	1

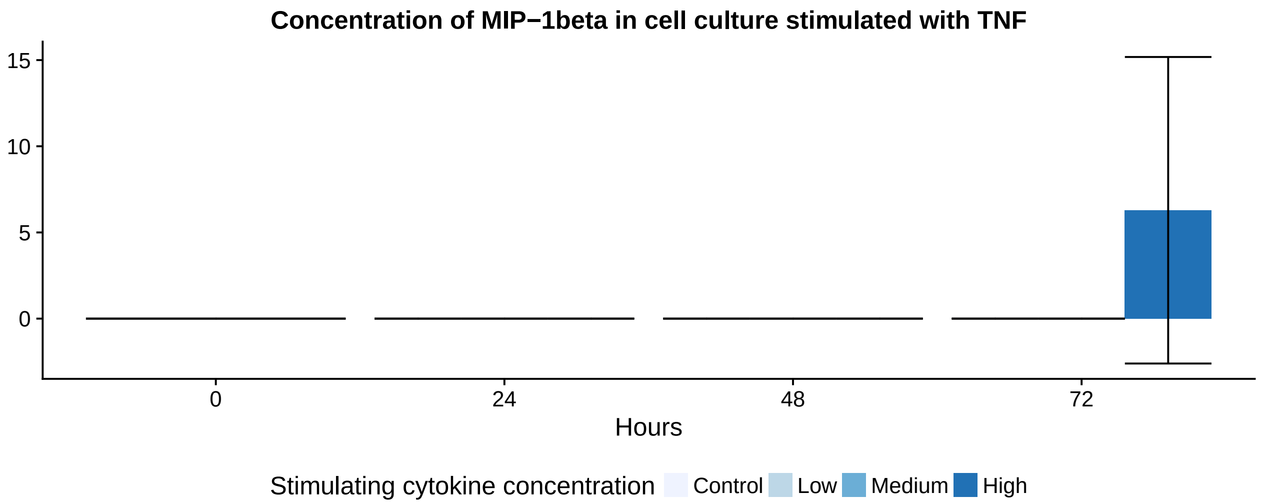

	Df	Sum Sq	Mean Sq	F value	Pr(>F)
Time	1	11.861683	11.861683	2.2392344	0.1501624
Concentration	1	2.965421	2.965421	0.5598086	0.4630456
Time:Concentration	1	30.794754	30.794754	5.8133971	0.0256422
Residuals	20	105.944093	5.297205	NA	NA

	0 0	24 1	24 2	24 3	48 1	48 2	48 3	72 1	72 2
24 1	1.0000000	NA	NA	NA	NA	NA	NA	NA	NA
24 2	1.0000000	1.000000	NA	NA	NA	NA	NA	NA	NA
24 3	1.0000000	1.000000	1.000000	NA	NA	NA	NA	NA	NA
48 1	1.0000000	1.000000	1.000000	1.000000	NA	NA	NA	NA	NA
48 2	1.0000000	1.000000	1.000000	1.000000	1.000000	NA	NA	NA	NA
48 3	1.0000000	1.000000	1.000000	1.000000	1.000000	1.000000	NA	NA	NA
72 1	1.0000000	1.000000	1.000000	1.000000	1.000000	1.000000	1.000000	NA	NA
72 2	1.0000000	1.000000	1.000000	1.000000	1.000000	1.000000	1.000000	1.000000	NA
72 3	0.2666309	0.863443	0.863443	0.863443	0.863443	0.863443	0.863443	0.863443	0.863443

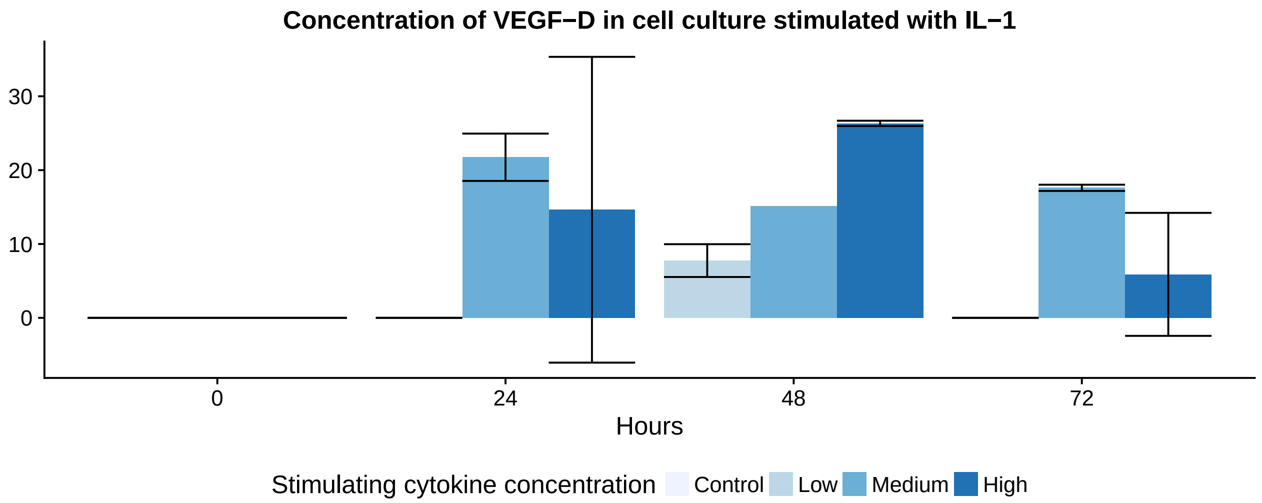

	Df	Sum Sq	Mean Sq	F value	Pr(>F)
Time	1	55.62504	55.62504	0.6878047	0.4191137
Concentration	1	863.48244	863.48244	10.6769774	0.0048388
Time:Concentration	1	37.94398	37.94398	0.4691781	0.5031686
Residuals	16	1293.97286	80.87330	NA	NA

	0 0	24 1	24 2	24 3	48 1	48 2	48 3	72 1	72 2
24 1	NA	NA	NA	NA	NA	NA	NA	NA	NA
24 2	NA	NA	NA	NA	NA	NA	NA	NA	NA
24 3	NA	NA	NA	NA	NA	NA	NA	NA	NA
48 1	NA	NA	NA	NA	NA	NA	NA	NA	NA
48 2	NA	NA	NA	NA	NA	NA	NA	NA	NA
48 3	NA	NA	NA	NA	NA	NA	NA	NA	NA
72 1	NA	NA	NA	NA	NA	NA	NA	NA	NA
72 2	NA	NA	NA	NA	NA	NA	NA	NA	NA
72 3	NA	NA	NA	NA	NA	NA	NA	NA	NA

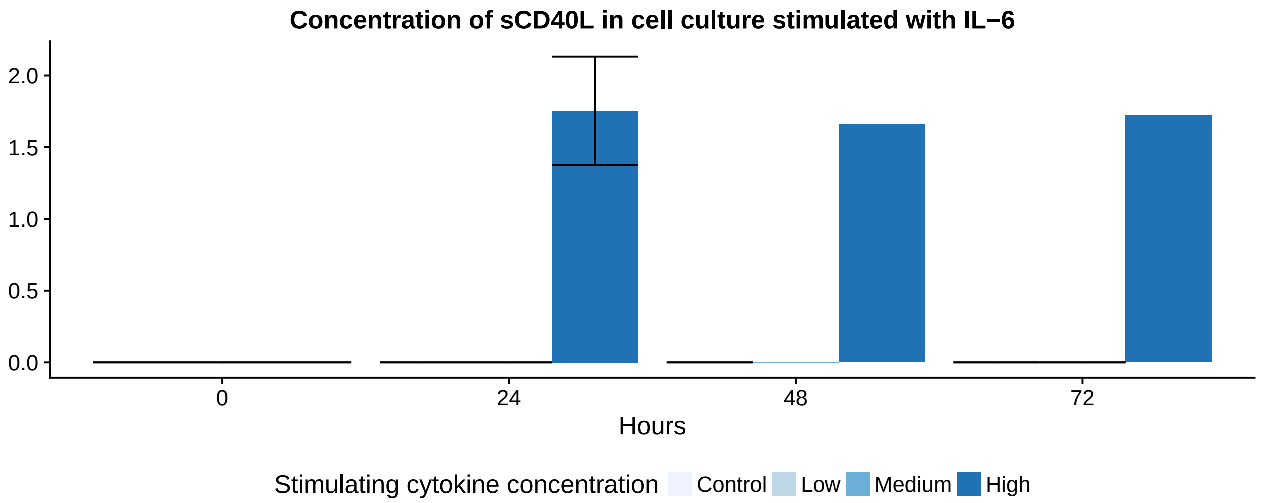

	Df	Sum Sq	Mean Sq	F value	Pr(>F)
Time	1	0.0393654	0.0393654	0.1835754	0.6748371
Concentration	1	5.9998030	5.9998030	27.9792993	0.0001143
Time:Concentration	1	0.3380938	0.3380938	1.5766562	0.2297958
Residuals	14	3.0021210	0.2144372	NA	NA

	0 0	24 1	24 2	24 3	48 1	48 2	48 3	72 1	72 2
24 1	NA	NA	NA	NA	NA	NA	NA	NA	NA
24 2	NA	NA	NA	NA	NA	NA	NA	NA	NA
24 3	NA	NA	NA	NA	NA	NA	NA	NA	NA
48 1	NA	NA	NA	NA	NA	NA	NA	NA	NA
48 2	NA	NA	NA	NA	NA	NA	NA	NA	NA
48 3	NA	NA	NA	NA	NA	NA	NA	NA	NA
72 1	NA	NA	NA	NA	NA	NA	NA	NA	NA
72 2	NA	NA	NA	NA	NA	NA	NA	NA	NA
72 3	NA	NA	NA	NA	NA	NA	NA	NA	NA
