# Supplementary material for: Delineating Astrocytic Cytokine Responses in a Human Stem Cell Model of Neural Trauma
Source: J Neurotrauma. 2019 Dec 11;37(1):93–105. doi: 10.1089/neu.2019.6480 (PMC6921298; doi:10.1089/neu.2019.6480)
Supplement: Supplemental data [file Supp_FigS3.pdf]

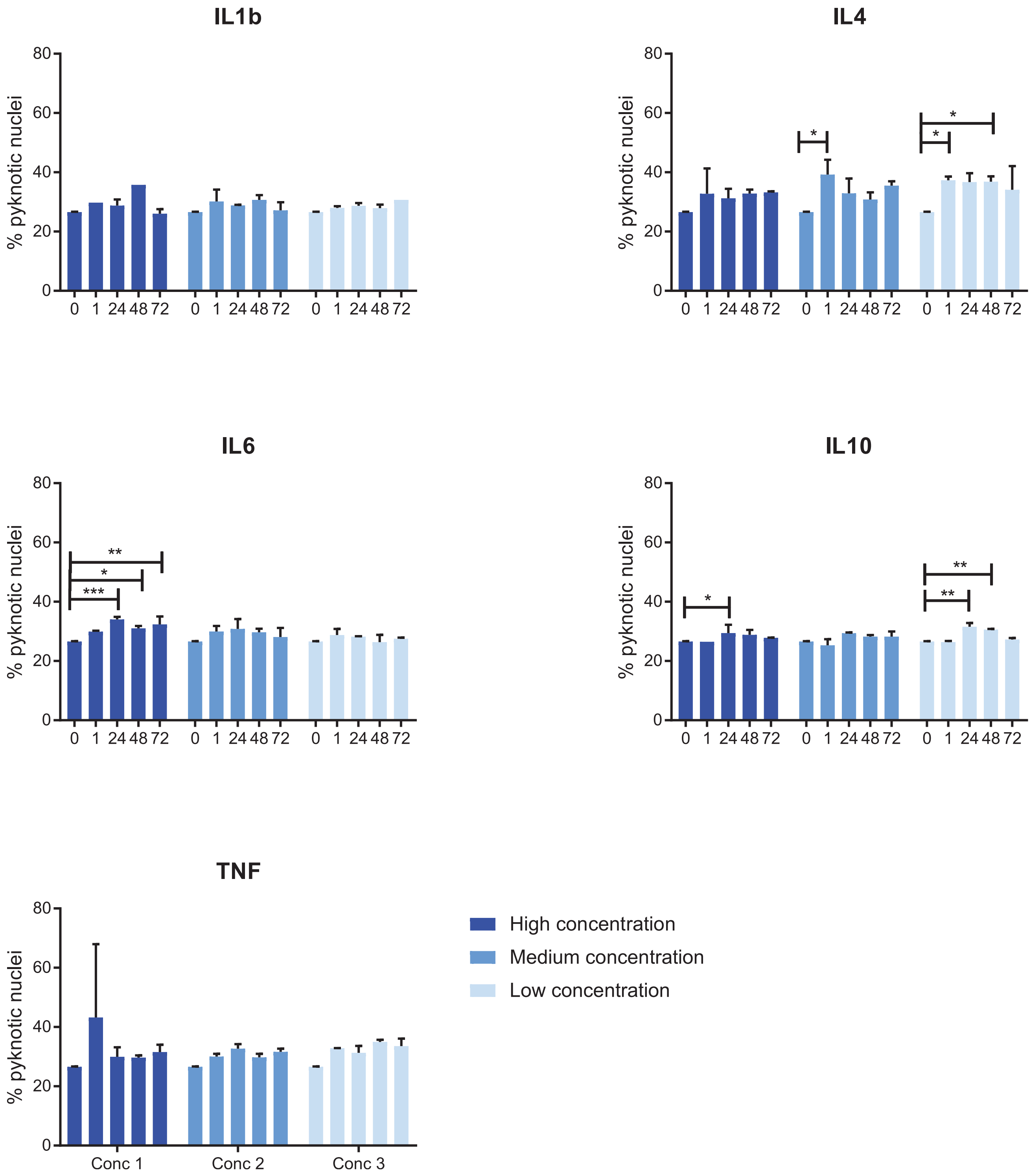

SUPPLEMENTARY FIG. S3. Bar graphs showing the proportion of pyknotic nuclei following treatments with different cytokine treatments. Pyknotic nuclei were automatically identified with DAPI staining based on the following parameters: nucleus area <40 μm^2, DAPI staining intensity >10000. Pyknotic nuclei were quantified at the following time points: 1, 24, 48, and 72 h. Three different concentrations were tested for each cytokine. Data shown are mean ± standard deviation (SD) from two technical replicates. Two way analysis of variance (ANOVA) with Dunnett's multiple comparison test: $*p < 0.05$, $**p < 0.01$, $***p < 0.001$. > 5000 cells per condition were analyzed.
