# Supplementary material for: Delineating Astrocytic Cytokine Responses in a Human Stem Cell Model of Neural Trauma
Source: J Neurotrauma. 2019 Dec 11;37(1):93–105. doi: 10.1089/neu.2019.6480 (PMC6921298; doi:10.1089/neu.2019.6480)
Supplement: Supplemental data [file Supp_FigS4.pdf]

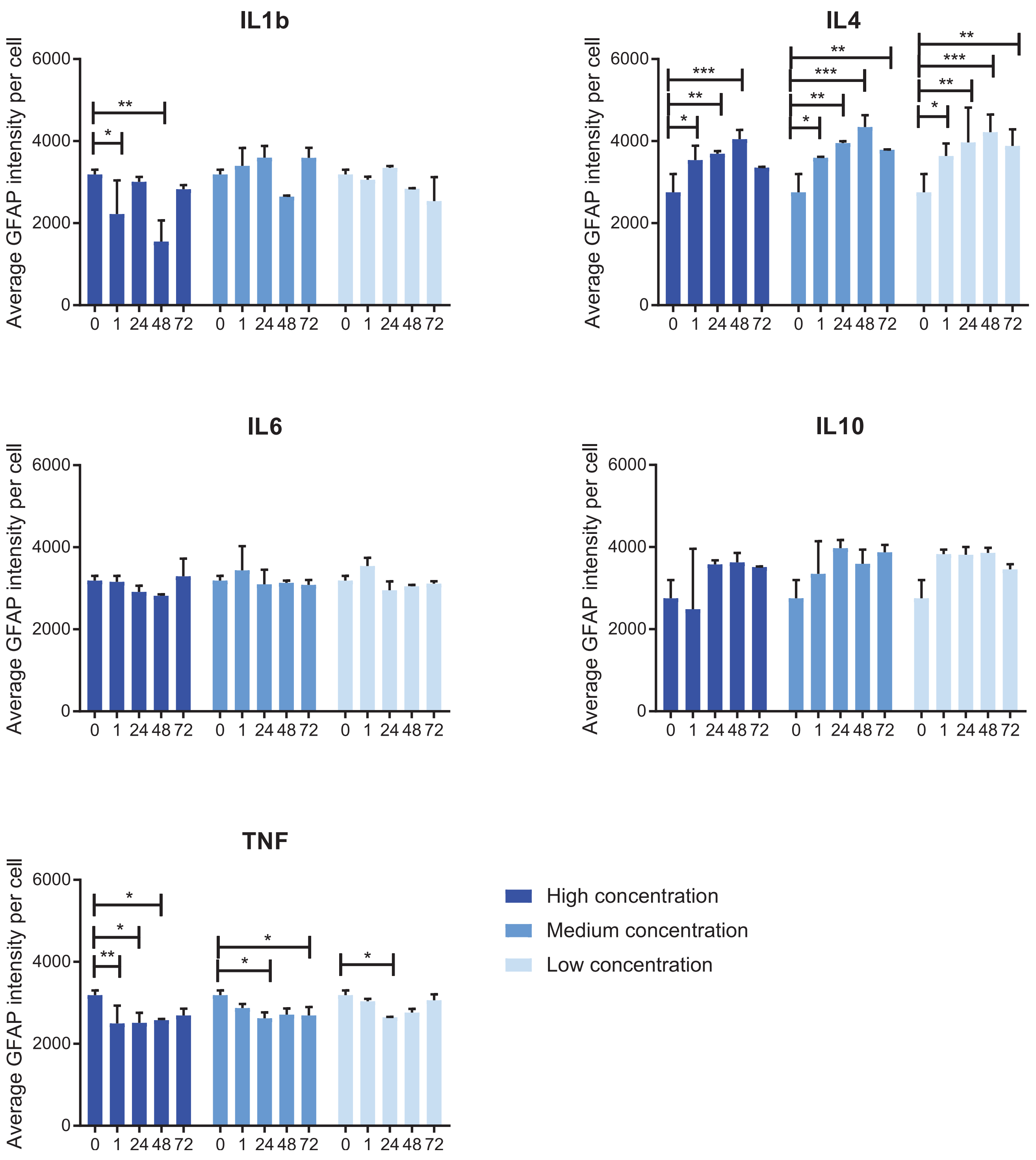


**SUPPLEMENTARY FIG. S4.** Bar graphs showing the average intensity of glial fibrillary acidic protein (GFAP) immunostaining per cell. Time points analyzed were 1, 24, 48, and 72 h. Three different concentrations were tested for each cytokine. Data shown are mean ± standard deviation (SD) from two technical replicates. Two way analysis of variance (ANOVA) with Dunnett's multiple comparison test: *$p<0.05$, **$p<0.01$, $p<0.001$. > 5000 cells per condition were analyzed.
